# TCF-ALP: a fluorescent probe for the selective detection of *Staphylococcus* bacteria and application in “smart” wound dressings†

**DOI:** 10.1039/d0bm01918f

**Published:** 2021-05-11

**Authors:** Lauren Gwynne, George T. Williams, Kai-Cheng Yan, Bethany L. Patenall, Jordan E. Gardiner, Xiao-Peng He, Jean-Yves Maillard, Tony D. James, Adam. C. Sedgwick, A. Toby. A. Jenkins

**Affiliations:** Department of Chemistry, University of Bath BA2 7AY UK a.t.a.jenkins@bath.ac.uk; School of Physical sciences, University of Kent CT2 7NH UK; Key Laboratory for Advanced Materials and Joint International Research Laboratory of Precision Chemistry and Molecular Engineering, Feringa Nobel Prize Scientist Joint Research Center, School of Chemistry and Molecular Engineering, Frontiers Center for Materiobiology and Dynamic Chemistry, East China University of Science and Technology 130 Meilong Road Shanghai 200237 P. R. China; School of Pharmacy and Pharmaceutical Sciences, Cardiff University CF10 3NB UK; School of Chemistry and Chemical Engineering, Henan Normal University Xinxiang 453007 China; Department of Chemistry, The University of Texas at Austin 105 E 24^th^ St A5300 Austin Texas 78712-1224 USA

## Abstract

Alkaline phosphatase (ALP) is an important enzyme-based biomarker present in several bacterial species; however, it is currently undervalued as a strategy to detect pathogenic bacteria. Here, we explore our ALP-responsive colorimetric and fluorescent probe (**TCF-ALP**) for such applications. **TCF-ALP** displayed a colorimetric and fluorescence response towards *Staphylococcus aureus* (*S. aureus*), with a limit of detection of 3.7 × 10^6^ CFU mL^−1^ after 24 h incubation. To our surprise, **TCF-ALP** proved selective towards *Staphylococcus* bacteria when compared with *Enterococcus faecalis* (*E. faecalis*), and Gram-negative *P. aeruginosa* and *E. coli*. Selectivity was also seen in clinically relevant *S. aureus* biofilms. Owing to the high prevalence and surface location of *S. aureus* in chronic wounds, **TCF-ALP** was subsequently encapsulated in polyvinyl alcohol (PVA)-based hydrogels as a proof-of-concept “smart” wound dressing. **TCF-ALP** hydrogels were capable of detecting *S. aureus* in planktonic and biofilm assays, and displayed a clear colour change from yellow to purple after 24 h incubation using *ex vivo* porcine skin models. Overall, **TCF-ALP** is a simple tool that requires no prior knowledge, training, or specialist equipment, and has the potential to overcome issues related to invasive swabbing and tissue biopsy methods. Thus, **TCF-ALP** could be used as a tool to monitor the early development of infection in a wound and allow for the rapid provision of appropriate treatment for *Staphylococcal* bacterial infections.

## Abstract 2

The optical response of an alkaline phosphatase (ALP) probe was evaluated against various bacterial species.

## Graphical abstract

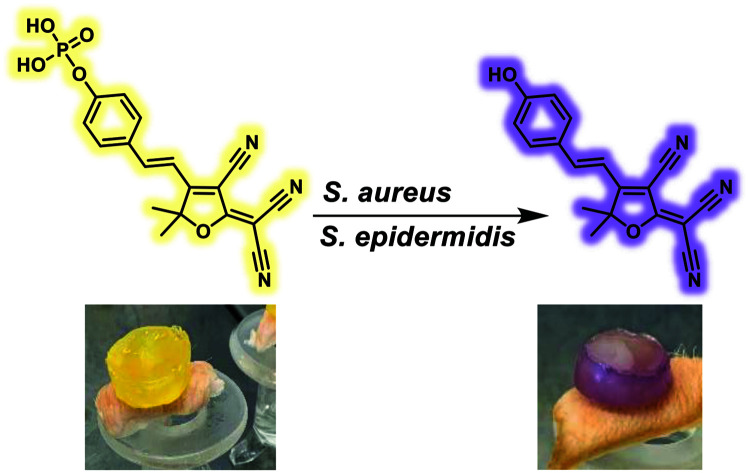

## Introduction

Chronic wounds affect the lives of millions worldwide and exert significant financial pressure on healthcare systems, with the NHS spending an estimated £ 4–5 billion per year.^1,2^ These non-healing wounds can persist for prolonged periods of time (months to years), often affecting patients with underlying health conditions such as diabetes, obesity, and cancer.^1,2^ Bacterial infections contribute significantly to the non-healing nature of these wounds with bacteria often forming complex bacterial communities known as biofilms.^3^ Protected by extracellular polymeric substances (EPS), biofilms result in hard-to-treat infections, abet in the development of antimicrobial resistance, and abet inflammation at the wound site, resulting in further tissue damage.^6,7^ If left untreated, these localised infections can develop into life-threatening systemic infections.^8,9^ As a result, effective wound management combined with the ability to rapidly identify and treat pathogenic bacteria is highly desirable for the treatment of chronic wounds.

Swab and tissue biopsy methods are currently the gold standard used to confirm the presence of a wound infection and used to identify the infection-causing pathogenic bacteria.^10,11^ However, these invasive and painful techniques have inherent limitations, requiring time-consuming protocols, highly trained specialists, and are limited to bacterial species that can be routinely grown in a laboratory.^10,12^ These limitations result in delayed treatment, potentially leading to a worse prognosis for the patient.^4^ Recent technological advancements have led to the development of assays that utilise genomic markers to rapidly identify pathogenic bacteria (ELISA, PCR, DNA microarrays and optical and electrochemical biosensors).^5–7,8–12^ However, as with conventional methods, most of these techniques are expensive and require extensive sample manipulation by trained specialists.^13–15^

**Scheme 1:**
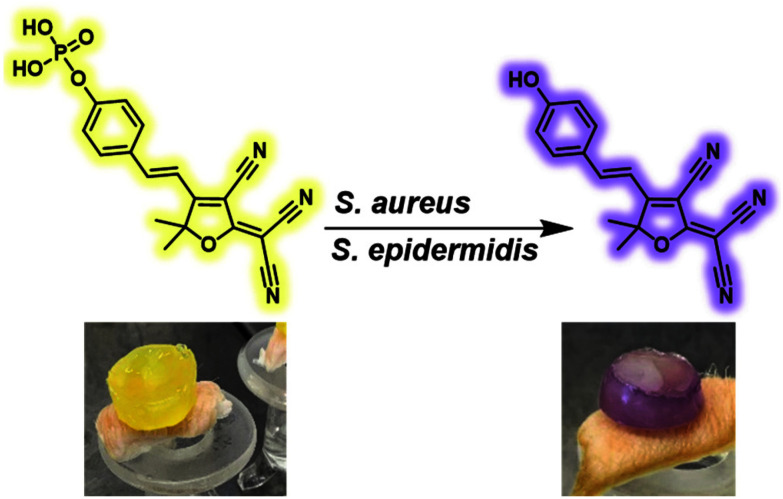
The use of **TCF-ALP** for the selective detection of *Staphylococcus* spp. *via* alkaline phosphatase dephosphorylation. Images show an *ex vivo* experiment utilising a PVA-hydrogel encapsulated with **TCF-ALP** before and after *S. aureus* NCTC 10788 inoculation.

Small molecule fluorescent and colorimetric probes provide an attractive alternative as they enable non-specialist use, are easy to handle and store, and confer a high degree of selectivity and sensitivity towards bacterial-based biomarkers.^16–22^ In addition, they offer a complementary strategy to smart wound technologies and point of care (PoC) devices.^23,24^ Amongst the bacterial based biomarkers, alkaline phosphatase (ALP) is an enzyme responsible for the hydrolysis of phosphoesters to facilitate the release of inorganic phosphate (Pi), an essential nutrient for bacterial cell growth.^25^ ALP is present in numerous bacteria species, including the ESKAPE pathogens, *Escherichia coli (E. coli)*,^26^*Pseudomonas aeruginosa* (*P. aeruginosa*),^27^ and *Staphylococcus aureus* (*S. aureus*).^28^ Therefore, it is not surprising that the determination of ALP activity has been a focal point for numerous microbiological studies,^29,30^ and as a strategy for the development of ALP-responsive small-molecule probes for the *in vitro* detection of bacteria.^31–33^ However, in comparison to other enzyme-based bacterial biomarkers, this enzyme has been undervalued as a strategy to detect pathogenic bacteria using small molecule fluorescent probes. In addition, recent studies have failed to develop viable platforms with the potential for clinical applications. In this work, we evaluated the ability of our previously designed ALP responsive fluorescent probe **TCF-ALP** to detect pathogenic bacteria (Scheme 1).^34^**TCF-ALP** was shown to selectively detect planktonic *S. aureus* and *Staphylococcus epidermidis* (*S. epidermidis*), and was effective in detecting *S. aureus* in a series of planktonic, biofilm, and *ex vivo* studies. Our work clearly demonstrates the use of **TCF-ALP** as a simple tool that could be used to monitor the early development of infection and allow for the rapid provision of the appropriate treatment for *Staphylococcal* bacterial infections. It is important to note that in this study, only clinically relevant *Staphylococcus* species (*S. aureus* and *S. epidermidis*^35^) were used with **TCF-ALP**.

## Results and discussion

**Fig. 1:**
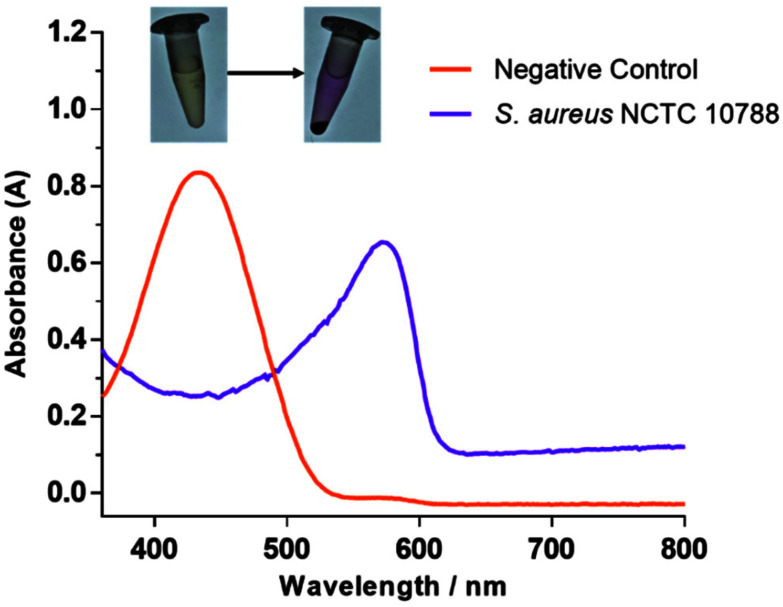
UV-Vis spectra of **TCF-ALP** (10 μM) after 24 h incubation at 32 °C with *S. aureus* NCTC 10788 (10^8^ CFU mL^−1^) in 50 mM Tris-HCl buffer, pH = 9.2.

Gram-positive *S. aureus* is often one of the first bacteria to establish within a wound, primarily colonising close to the surface.^36^ In comparison, Gram-negative *P. aeruginosa* is a late-stage coloniser of chronic wounds and is predominately found in the deeper regions of the wound.^36^ Therefore, the surface location and high prevalence of *S. aureus* represents a unique opportunity to be used for the early diagnosis of chronic wound development. Hence, with this work, *S. aureus* NCTC 10788 was chosen for evaluating the ability of **TCF-ALP** as a diagnostic tool. Initial experiments revealed that incubation of **TCF-ALP** (10 μM) with *S. aureus* NCTC 10788 (10^8^ CFU mL^−1^) for 24 h led to a bathochromic shift in UV-Vis absorption and an easy-to-visualise colour change from yellow to purple (Fig. 1). This result was indicative of the ALP-mediated transformation of **TCF-ALP** to **TCF-OH**.

For all experiments, *S. aureus* NCTC 10788 was grown for 24 h at 32 °C in Mueller Hinton broth (MH; pH 7.4) before centrifugation and resuspension in a solution of **TCF-ALP** (10 μM, 50 mM Tris HCl, pH 9.2). The resuspended solution was held at pH of 9.2 since this previously has been shown to be optimum for ALP activity.^34^ MH broth was chosen over other non-differential broths such as Tryptic Soy Broth (TSB) and Luria–Bertani (LB) as it provided the greatest fluorescence response (Fig. S1 and S2†). The difference in response was attributed to the varying amounts of inorganic phosphate (Pi) found in each medium, which can serve as an ALP inhibitor.^37^ Optimisation experiments ensured no interference or unwanted response occurred from residual growth medium (*S. aureus* bacterial pellets were washed 1 to 3 times with Tris-HCl buffer [50 mM, pH 9.2] – Fig. S3†).

**Fig. 2:**
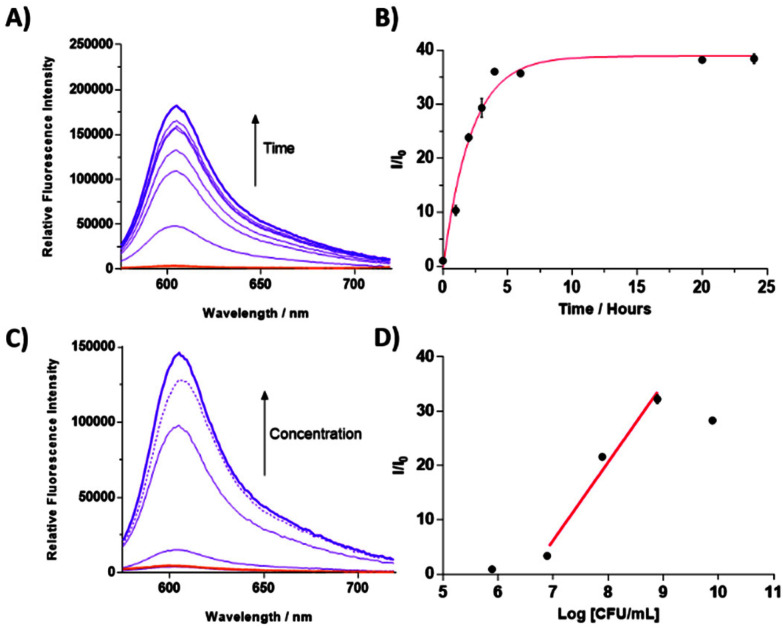
(A) Fluorescence spectra of **TCF-ALP** (10 μM) recorded over the course of 24 h upon addition of *S. aureus* NCTC 10788 (10^8^ CFU mL^−1^) in 50 mM Tris-HCl buffer pH = 9.2 at 32 °C. (B) Corresponding change in fluorescence (*I*/*I*_0_) of **TCF-ALP** (10 μM) upon addition of *S. aureus* NCTC 10788 (10^8^ CFU mL^−1^) in 50 mM Tris-HCl buffer pH = 9.2 at 32 °C. Error bars indicate standard deviation (*n* = 3). (C) Fluorescence spectra of **TCF-ALP** (10 μM) after 24 h incubation with various concentrations of *S. aureus* NCTC 10788 (0–10^9^ CFU mL^−1^) in 50 mM Tris-HCl buffer pH = 9.2 at 32 °C with the dotted line representing 10^10^ CFU mL^−1^, and (D) corresponding sensitivity graph. (X-intercept 6.568 = 3.70 × 10^6^ CFU mL^−1^; *Y* = 14.45*X*–94.88; *R*^2^ 0.9759). All experiments used *λ*_ex_ = 542 (bandwidth 15) nm and *λ*_em_ = 606 nm.

Given that our overall goal was to use **TCF-ALP** in wound dressing/diagnostic applications, we investigated the overall fluorescence response of **TCF-ALP** towards *S. aureus* under varying incubation temperatures (25 °C – room temperature, 32 °C – surface temperature of skin and 37 °C – optimum growth conditions) (Fig. S4†). Marginal differences were observed for the overall fluorescence responses when **TCF-ALP** was incubated with *S. aureus* at each of these temperatures, demonstrating the suitability of the system for use as a PoC device or in smart wound applications. Following on from our initial studies, fluorescence analysis was performed to determine the minimum incubation time needed for *S. aureus* NCTC 10788 (10^8^ CFU mL^−1^) to generate a response (Fig. 2A and B). After 1 h incubation, an approximate 10-fold increase in fluorescence intensity was observed, which continued to increase to a >30-fold increase in fluorescence intensity after approximately 10 h.

Next, **TCF-ALP** (10 μM) was incubated with varying concentrations of *S. aureus* NCTC 10788 (10^5^–10^10^ CFU mL^−1^), and the fluorescence intensity was recorded after 1 h and 24 h incubation. After 1 h incubation, a linear fluorescence increase was observed for bacterial concentrations between 8–10 log CFU mL^−1^ (Fig. S5†). The LOD, as determined by linear regression, was found to be 3.17 × 10^7^ CFU mL^−1^ (95% confidence interval (CI) 2.95–3.40 × 10^7^ CFU mL^−1^). After 24 h incubation, a linear fluorescence increase was observed for bacterial concentrations between 7–9 log CFU mL^−1^ (Fig. 2C and D). A slight decline in the fluorescence response was observed for 10^10^ CFU mL^−1^ of *S. aureus* NCTC 10788; this is believed to be the result of the cellular uptake of **TCF-ALP** at high bacterial concentrations. This hypothesis was supported through fluorescence imaging studies (see ESI – Fig. S6 and S7†). At 24 h, the LOD was determined as 3.7 × 10^6^ CFU mL^−1^ (95% CI of 2.08–5.79 × 10^6^ CFU mL^−1^). This LOD is in line with other fluorescent and colorimetric probes used for the detection of a variety of bacterial enzymes (Table S2†). Biofilms are known to have a significantly higher bacterial cell density compared to the concentration of planktonic bacteria thought to result in a localised infection (10^5^ CFU mL^−1^).^38^ As a result, we believe the high LOD of **TCF-ALP** is advantageous, as the clear “turn-on” fluorescence and colorimetric response is witnessed at infection-related concentrations (>10^6^ CFU mL^−1^). Fluorescent/colorimetric probes with a LOD below these values may lead to false positive results (Table S2†).

### Bacterial selectivity of **TCF-ALP**

**Fig. 3:**
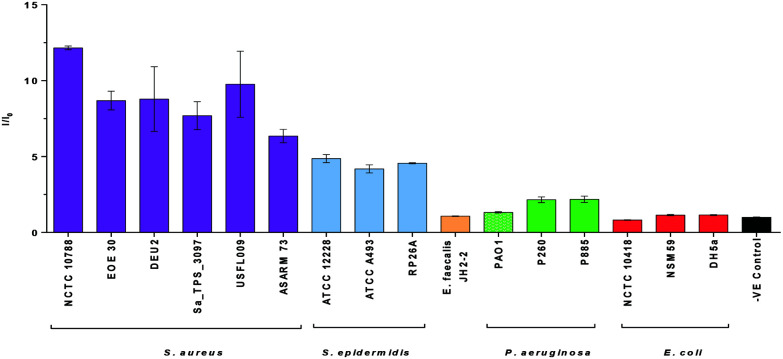
Selectivity bar chart of **TCF-ALP** (10 μM) in 50 mM Tris-HCl buffer pH = 9.2 after 1 h incubation with various bacterial strains (10^8^ CFU mL^−1^) at 32 °C. *λ*_ex_ = 542 (bandwidth 15) nm. *λ*_em_ = 606 nm. Error bars indicate standard deviation (*n* = 3).

Diagnostic devices that selectively detect specific types of bacteria are particularly attractive as they would allow for the rapid provision of the appropriate treatment to improve prognosis and avoid the misuse of antibiotics. This led us to perform selectivity studies for **TCF-ALP** against various bacterial species. In this study, we initially evaluated the fluorescence response of **TCF-ALP** against six Gram-positive *S. aureus* strains, three *S. epidermidis* strains, one Gram-positive *Enterococcus faecalis (E. faecalis*) strain, and three bacterial strains of Gram-negative *P. aeruginosa* and *E. coli*. All isolates used in this study were either relevant clinical isolates or reference strains, with the province outlined in the ESI – see Methods (Table S1†). As seen in Fig. 3, all *Staphylococcal* strains displayed a clear fluorescence turn-on response. However, Gram-positive *E. faecalis* and all Gram-negative bacteria tested resulted in a negligible increase in fluorescence intensity, even after 24 h (see ESI – Fig. S8†). Cell counting confirmed that **TCF-ALP** had a minimal effect on Gram-negative bacterial cell viability (Fig. S9†). Additionally, the commercially available colorimetric ALP probe, *p*-nitrophenyl phosphate (*p*-NPP), was used to determine ALP activity in each bacterial species. While *S. aureus*, *E. faecalis* and *E. coli* were shown to have similar ALP activities, minimal ALP activity was observed for *P. aeruginosa* (Fig. S10 and S11†). These experiments further suggested the selectivity of **TCF-ALP** towards *Staphylococcal* species. To further illustrate this selectivity, **TCF-ALP** was then evaluated against a total of 42 *S. aureus* isolates. Remarkably, all *S. aureus* strains produced at least a 10-fold increase in fluorescence intensity after 24 h, regardless of the phenotype (Fig. S12 and S13†). This demonstrates the potential use of **TCF-ALP** as a tool for the rapid detection of *S. aureus* in a clinical setting.

### Detection of ALP within *S. aureus* biofilms

As bacterial biofilms are present in the majority of infected chronic wounds,^6,7^ we turned our attention towards the utility of **TCF-ALP** to respond to *S. aureus* biofilms. Remarkably, when 24 h old *S. aureus* NCTC 10788 biofilms (formed in 96-well microtiter plates) were incubated with **TCF-ALP** (10 μM) for 24 h at 32 °C, we observed up to a 40-fold increase in fluorescence intensity (Fig. S14†). ALP inhibition experiments were performed to ensure that ALP activity was responsible for the fluorescence “turn-on” of **TCF-ALP**. Upon increasing concentrations of a known inhibitor (Sodium orthovanadate) a decrease in fluorescence intensity was observed, confirming the ALP-mediated hydrolysis of **TCF-ALP** (Fig. S15 and S16†).

**Fig. 4:**
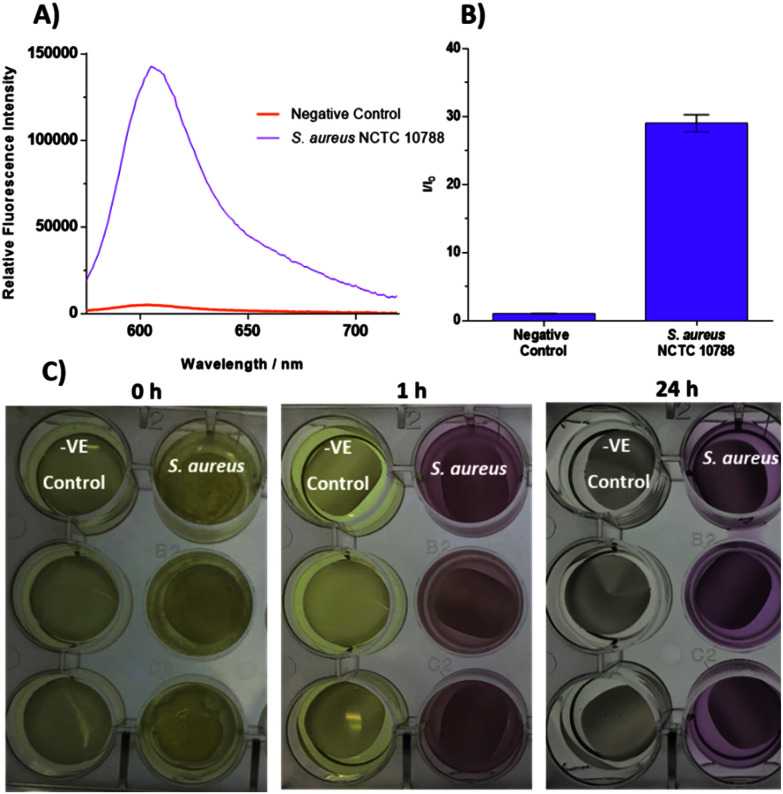
(A) Fluorescence spectra of **TCF-ALP** (10 μM) after 24 h incubation with biofilms of *S. aureus* NCTC 10788 (10^11^ CFU per membrane) in 50 mM Tris-HCl buffer pH = 9.2 at 32 °C. (B) Corresponding selectivity bar chart. *λ*_ex_ = 542 (bandwidth 15) nm. *λ*_em_ = 606 nm. Error bars indicate standard deviation (*n* = 3). (C) Images taken of negative controls (Membrane and Artificial Wound Fluid (AWF) only) and biofilms of *S. aureus* NCTC 10788 (10^11^ CFU per membrane) after 0, 1, and 24 h incubation with 10 μM **TCF-ALP** in 50 mM Tris-HCl buffer pH = 9.2 at 32 °C.

With these promising results in hand, a more robust biofilm model was used, termed the colony biofilm model; this provides a more accurate representation of a biofilm in a clinical setting.^39^ Colony biofilms were grown on a permeable polycarbonate membrane on a MH agar plate – used to mimic the surface of an infection site. A representative wound environment was achieved by treating the polycarbonate membranes with artificial wound fluid (AWF; 50% fetal bovine serum in 50% peptone water [0.9% sodium chloride in 0.1% peptone]) before the addition of bacteria. As seen in Fig. 4A and B, a large increase in fluorescence intensity was observed when *S. aureus* NCTC 10788 biofilms were incubated with **TCF-ALP** (10 μM), compared to the negative control. A positive turn-on response and a colour change from yellow to purple also occurred after only 1 h incubation with **TCF-ALP** (Fig. 4C and S17†). Conversely, minimal fluorescence responses were observed when **TCF-ALP** was incubated with Gram negative bacterial biofilms, further highlighting the selectivity of this probe (Fig. S18–S20†) **TCF-ALP** also had minimal effect on the bacterial viability of all bacterial biofilms tested (Fig. S21†).

### Incorporation of TCF-ALP into polyvinyl alcohol-based (PVA) hydrogels and use in the detection of *S. aureus*

**Fig. 5:**
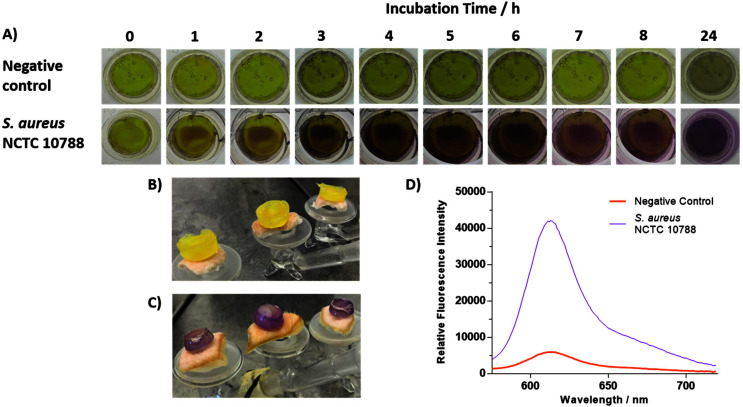
(A) Images depicting the colour of **TCF-ALP**-based PVA hydrogels in the presence of a negative control (Membrane and Artificial Wound Fluid (AWF) only) and a *S. aureus* NCTC 10788 biofilm (10^11^ CFU per membrane). Images taken hourly at 0–8 h and 24 h and were repeated in triplicate. (B) **TCF-ALP**-based PVA hydrogels on non-inoculated porcine skin wound model after 24 h incubation. (C) **TCF-ALP**-based PVA hydrogels on a porcine skin wound model inoculated with *S. aureus* NCTC 10788 (10 μL of 10^8^ CFU mL^−1^), and (D) corresponding fluorescence intensities of the hydrogels shown in (B) and (C). *λ*_ex_ = 542 (bandwidth 15) nm. *λ*_em_ = 606 nm.

As previously mentioned, traditional methods of bacterial detection utilise swabbing and tissue biopsy. A major drawback to these techniques is the requirement for the removal of a conventional wound dressing. This has the potential to damage healing tissue, can cause pain to the patient, and risk exposure to infection.^40^ Hydrogels are being increasingly used in the field of wound care as they maintain a moist wound environment, accelerate the healing processes, are easy to remove, and are easy to develop and handle.^41^ However, only a few diagnostic hydrogel systems have currently been designed for the detection of bacteria.^26,29^ To demonstrate the potential of **TCF-ALP** for use in “smart” wound dressing systems,^42^ we turned our attention to the encapsulation of **TCF-ALP** within a PVA-based hydrogel. In brief, **TCF-ALP** (38.5 μL, 100 μM in DMSO) was suspended in 1 mL of 10% w/v PVA (50 mM Tris HCl, pH 9.2). This solution was then subjected to a single freeze–thaw cycle, producing a mechanically stable hydrogel. To account for any potential loss of probe during the freeze–thaw cycle, **TCF-ALP** was loaded into the hydrogels at a higher concentration (100 μM). With **TCF-ALP** encapsulated hydrogels in hand, we evaluated their ability to respond to planktonic *S. aureus* NCTC 10788. Each hydrogel was incubated in 2 mL of a 10^8^ CFU mL^−1^*S. aureus* suspension in 50 mM Tris HCl (pH 9.2) and photographed at regular time intervals (Fig. S22†). After 5 h, the hydrogels exhibited a significant colour change from yellow to purple, progressing to a deep purple after 24 h incubation, and a strong corresponding fluorescence intensity at 606 nm — indicative of the formation of **TCF-OH** (Fig. S23†). While it is important to note that small amounts of **TCF-ALP** leached from the system, this is a proof-of-concept study with the opportunity to optimise the system at a later stage of development. Experiments with **TCF-ALP**-based hydrogels using *S. aureus* NCTC 10788 colony biofilms displayed a remarkable colour change from yellow to purple (Fig. 5A), with a concomitant increase in fluorescence intensity at 606 nm (Fig. S24†). Interestingly, the location on the hydrogel in which the change in colour gradually occurred was the contact point between the hydrogel and bacterial surface. Additionally, both the blank hydrogel and **TCF-ALP** hydrogel had no overall effect on the viability of *S. aureus* NCTC 10788 (Fig. S25†).

### *Ex vivo* porcine skin models

Finally, to demonstrate the potential diagnostic capability of **TCF-ALP**, *ex vivo* skin models were performed using porcine skin, owing to its similarity to human skin.^43^ In this *ex vivo* study, porcine skin was treated with 10 μL of *S. aureus* NCTC 10788 (10^8^ CFU mL^−1^) and allowed to dry at room temperature. Subsequent 24 h incubation of the *S. aureus*-treated porcine skin with 1 mL of **TCF-ALP** (10 μM. 50 mM Tris HCL, pH 9.2) resulted in an 8-fold increase in fluorescence intensity, with an associated colour change from yellow to purple; no overall change to cell concentration was observed (Fig. S26–S28†). Additionally, **TCF-ALP** hydrogels were evaluated using the *ex vivo* model in an attempt to model a clinically relevant situation. To our excitement, **TCF-ALP** hydrogels placed on *S. aureus*-treated porcine skin resulted in a clear colour change from yellow to purple after 24 h incubation. In addition, this colour change corresponded to an approximate 7-fold increase in fluorescence intensity (Fig. 5B–D), with no difference in bacterial concentration observed (Fig. S29†). As such, we believe these results demonstrate the potential of **TCF-ALP** to be used in the development of smart wound dressings for the rapid identification of an infected wound to provide the appropriate treatment without removal of the patient's wound dressing.

## Conclusion

Rapid detection of bacterial species present within a wound is important for the development of suitable and rapid treatment protocols. New methods that allow for quick PoC detection are thus of great importance, especially as we continue to enter the era of antibiotic resistance. With this aim in mind, the ALP-responsive colorimetric and fluorescent probe **TCF-ALP** was evaluated for its response to pathogenic bacteria. In the presence of Gram-positive *S. aureus* NCTC 10788, **TCF-ALP** was shown to have an excellent colorimetric and fluorescence response with a limit of detection of 3.7 × 10^6^ CFU mL^−1^ after 24 h incubation. To our surprise and delight, **TCF-ALP** proved selective towards *S. aureus* and *S. epidermidis* compared to one Gram-positive *E. faecalis* strain and six Gram-negative strains (three *P. aeruginosa* and three *E. coli*) with selectivity seen in clinically relevant biofilm models. **TCF-ALP** was then evaluated against a total of 42 *S. aureus* isolates. Remarkably, all 42 *S. aureus* strains produced at least a 10-fold increase in fluorescence intensity after 24 h, regardless of the phenotype. **TCF-ALP** was encapsulated in PVA-based hydrogels as a proof of concept for “smart” wound dressing applications. **TCF-ALP** hydrogels proved effective for the detection of *S. aureus* as planktonic and biofilm bacteria. *Ex vivo* skin models with **TCF-ALP** hydrogels resulted in a clear colour change from yellow to purple after 24 h incubation. Overall, **TCF-ALP** represents a simple diagnostic tool that requires no prior knowledge, no training/specialist equipment, and overcomes the time consuming and invasive swabbing and tissue biopsy methods. Thus, **TCF-ALP** could be used as a tool to monitor the early development of infection in a wound and allow the rapid provision of the appropriate treatment for *Staphylococcal* bacterial infections.

## Conflicts of interest

There are no conflicts of interest to declare.

## Supplementary Material

BM-009-D0BM01918F-s001
