# Supplementary material for: TCF-ALP: a fluorescent probe for the selective detection of Staphylococcus bacteria and application in “smart” wound dressings
Source: Biomater Sci. 2021 May 11;9(12):4433–9. doi: 10.1039/d0bm01918f (PMC8204156; doi:10.1039/d0bm01918f)
Supplement: BM-009-D0BM01918F-s001 [file BM-009-D0BM01918F-s001.pdf]

Electronic Supplementary Material (ESI) for Biomaterials Science.
This journal is © The Royal Society of Chemistry 2021

Supplementary Information

TCF-ALP: A fluorescent probe for the selective detection of *Staphylococcus* bacteria and application in "smart" wound dressings

Lauren Gwynne,[a] George T. Williams,[a,b] Kai-Cheng Yan,[c], Bethany L. Patenall,[a] Jordan E. Gardiner,[a] Xiao-Peng He,[c] Jean-Yves Maillard,[d] Tony D. James,[a, f*] Adam C. Sedgwick,[e*] and A. Toby A. Jenkins,*[a]

[a]Department of Chemistry, University of Bath, Bath, BA2 7AY, U.K.

[b]School of Physical Sciences, University of Kent, CT2 7NH, U.K.

[c]Key Laboratory for Advanced Materials and Joint International Research Laboratory of Precision Chemistry and Molecular Engineering, Feringa Nobel Prize Scientist Joint Research Center, School of Chemistry and Molecular Engineering, Frontiers Center for Materiobiology and Dynamic Chemistry, East China University of Science and Technology, 130 Mei-long Road, Shanghai 200237, P. R. China

[d]Cardiff School of Pharmacy and Pharmaceutical sciences, Cardiff University, Cardiff, U.K.

[e]Department of Chemistry, The University of Texas at Austin, 105 E 24th St. A5300, Austin, Texas, 78712-1224, U.S.A.

[f]School of Chemistry and Chemical Engineering, Henan Normal University, Xinxiang 453007, China.

Corresponding authors:

T.D.James@bath.ac.uk

A.T.A.Jenkins@bath.ac.uk

A.C.Sedgwick@utexas.edu

Table of Contents

1. Materials and Methods S3

2. Role of Bacterial Growth Media on ALP Production S10

3. Washing Steps S12

4. Incubation Temperatures S13

5. Limit of Detection S14

6. Fluorescence imaging of *Staphylococcus aureus* using Confocal Laser-Scanning Microscope (CLSM) S16

6. Additional Selectivity Experiments and Cell Count for Selectivity Assay S18

7. 96-well Microtiter Plate Biofilms S21

8. Sodium Orthovanadate Minimum Inhibitory Concentration and Minimum Biofilm Inhibition Concentration S22

9. Effect of Sodium Orthovanadate on ALP Production in *S. aureus* Biofilms S23

10. Colony Biofilm Models S24

11. ....TCF-ALP Hydrogels for Planktonic Bacterial Detection S27

12. ....TCF-ALP Hydrogels with Colony Biofilm Models of *S. aureus* S28

13. *Ex vivo* experiments using TCF-ALP S29

14. References S31

1. Materials and Methods

Synthesis of TCF-ALP

TCF-ALP was synthesised as outlined in *Gwynne et al*.[1] Stock solutions of **TCF-ALP** were stored in DMSO at 4 °C until required.

Preparation of TCF-ALP based PVA hydrogels

A 10% w/v PVA solution was prepared by dissolving poly vinyl alcohol (PVA) in 50 mM Tris HCl (pH 9.2). After the resultant solution had cooled to room temperature, aliquots of 1 mL were transferred into a 12-well microtiter plate and 38.5 µL of **TCF-ALP** (2.6 mM in DMSO) added to produce homogenous yellow solutions. These solutions were then placed at -80 °C and underwent one freeze-thaw cycle to produce mechanically stable hydrogels, which were protected from light and stored at 4 °C prior to use.

Bacterial Strains

S. aureus NCTC 10788 and *E. coli* NCTC 10418 were acquired from Public Health England (PHE; Porton Down, U.K). All further strains used in this assay were kindly provided by Professor Toby Jenkins, Dr Maisem Laabei and Dr Susanne Gebhard, University of Bath, U.K. A full list of bacterial strains used can be found in **Table S1**.

Table S1: List of bacterial strains used in this study.

Bacterial Strain	Comments	Reference
S. aureus NCTC 10788	PHE reference strain. Methicillin sensitive.	[2], [3]
S. aureus EOE30	Clinical MRSA strain isolated in 1999 from a patient admitted to Addenbrooke's hospital, Cambridge, UK, with their first episode of *Staphylococcus aureus* bacteraemia.	[4]
S. aureus DEU2	Clinical ST239 isolate from a catheter at Dokuz Eylul	[5], [6]

	University, Turkey.	
S, aureus Sa_TPS_3097	Unpublished isolate from the CC93 MRSA isolates.	[7]
S. aureus USFL009	Wildtype clinical MRSA USA300, isolated from nose/skin carriage.	[8], [9]
S. aureus ASARM73	Clinical strain isolated from a patient admitted to Addenbrooke's hospital, Cambridge, UK, with their first episode of *Staphylococcus aureus* bacteraemia.	[4]
S. epidermidis ATCC 12228	Non-biofilm forming, non-infection associated strain.	[10], [11], [12]
S. epidermidis A493	Unpublished *S. epidermidis* strain in Prof. Toby Jenkins research group	[13]
S. epidermidis RP26A	Methicillin-resistant, biofilm forming clinical isolate.	[14], [15]
P. aeruginosa PAO1	Reference strain, a mutant of the original PAO strain isolated in 1954 from a wound in Melbourne, Australia.	[16], [17]
P. aeruginosa P260	Isolated from an infected chronic wound, and acquired from AmpliPhi Biosciences (Bedfordshire, UK).	[18]
P. aeruginosa P885	Isolated from an infected chronic wound, and acquired from AmpliPhi Biosciences	[18]

	(Bedfordshire, UK).	
E. coli NCTC 10418	PHE reference strain, originally isolated in 1965.	[19]
E. coli NSM59	Clinical isolate from a UTI. Obtained by culture collection from the University of Brighton (Brighton, UK)	[20]
E. coli DH5α	Laboratory isolate developed by D. Hanahan for cloning use.	[21]
Enterococcus faecalis (*E. faecalis*) JH2-2	Clinical isolate from Hammersmith Hospital	[22]

Bacterial Growth Conditions

Bacterial strains were stored at –80 °C in broth containing 15% (v/v) glycerol until required. Working stocks of bacteria were prepared by streaking onto Tryptic Soy Agar (TSA) agar before incubation at 37 °C for 24 h. Plates were stored at 4 °C for up to a month for further use. Overnight cultures of bacterial strains were routinely propagated by transferring a single colony to 5 mL Mueller Hinton broth and incubating at 32 °C for 18 h. Cultures were washed via centrifugation (4000 g, 10 min) before being washed once and re-suspended in 50 mM Tris-HCl (pH 9.2) to an optical density at 600 nm of ~ 1 (c. 5 x 10^8 Colony Forming Units (CFU)/mL).

Bacterial Enumeration

Estimation of the total viable count of bacterial cultures were determined using the drop count method as outlined by Miles and Misra [23]. The initial bacterial suspension underwent a series of 10-fold dilutions in PBS, and subsequently three 10 µL spots of each dilution were pipetted onto the surface of a TSA agar plate. The spots were allowed to dry for 20 min at room temperature before incubation at 37 °C for 18 h. The number of Colony Forming Units per mL (CFU/mL) was calculated as follows:

$$CFU/mL = \frac{Number\ of\ colonies}{d \cdot V} \qquad (1)$$

Where d = dilution factor, and V = volume of inoculum (mL).

Detecting Alkaline Phosphatase in Planktonic Bacteria

Optically-adjusted bacterial cultures (**1.4**) underwent centrifugation (4000 g, 10 min) and were re-suspended in an equal volume of 10 µM **TCF-ALP** in 50 mM Tris-HCl (pH 9.2). Alternatively, for **TCF-ALP** hydrogel analysis, 2 mL of the bacterial culture in 50 mM Tris HCl (pH 9.2) was transferred to a 12-well microtiter plate containing a 100 µM **TCF-ALP** based PVA hydrogel. These suspensions were subsequently protected from light and incubated at 32 °C for 24 h, unless stated otherwise. For both assays, 1 mL was removed from each suspension and centrifuged at 10 000 g for 3 min. The supernatant was subsequently placed into a black or clear 96-well microtiter plate for fluorescence and UV-Vis analysis, respectively. The fluorescence was measured using CLARIOstar fluorimeter, λ_{ex} = 542 nm, λ_{em} = 606 nm, and the UV-Vis by SPECTROstar Omega. Appropriate controls were carried out in tandem and a minimum of three biological replicates per bacterial strain were used.

ALP Activity of 96-well Biofilms

Overnight cultures of *S.* aureus NCTC 10788 were sub-cultured into fresh Mueller Hinton broth to attain a concentration of 10^6 CFU/mL, before 200 µL aliquots were placed into a 96-well microtiter plate and incubated at 32 °C for 24 h. After incubation, planktonic bacteria were discarded, and the remaining biofilm was washed three times with sterile 50 mM Tris HCl (pH 9.2). Plates were left to dry at room temperature for 20 min, before subsequent addition of 220µL of 10 µM **TCF-ALP** in 50 mM Tris-HCl (pH 9.2) and further incubation at 32 °C for 24 h. From each well, a 200 µL aliquot was removed and centrifuged at 10 000 g for 3 min. The supernatant was subsequently placed into a black 96-well microtiter plate for fluorescence analysis of ALP production.

ALP activity of bacterial isolates using *p*-nitrophenyl phosphate

Prior to the determination of enzymatic activity, a calibration curve of *p*-nitrophenol (*p*-NP) was obtained by measuring the absorbance at 405 nm of increasing concentrations of *p*-NP (0 – 90 µM) in 50 mM Tris-HCl (pH 9.2) containing 250 mM NaOH.

Bacterial isolates (10^8 CFU/mL, 1 mL) were centrifuged (10,000g, 3 min) and resuspended in 950 µL of 10 mM *p*-nitrophenyl phosphate (*p*-NPP) in 50 mM Tris-HCl, pH = 9.2. After incubation at 32°C for 1 h, 50 µL of 5 M NaOH was added to stop the reaction. The resultant solution was centrifuged (10,000g, 3 min) and the absorbance of the supernatant was measured at 405 nm. The enzymatic activity of the solution was then determined by using the calibration curve of *p*-nitrophenol. 1 U (µmol/min) was defined as the amount of the enzyme that catalysed the conversion of one micromole of *p*-NPP per minute under the specified conditions of the assay method.

Role of Phosphatase Inhibitor on 96-well Biofilms

A 50 mM stock of sodium orthovanadate was prepared in deionised water (dH_2O). Once dissolved, the pH was adjusted to 9.2 with NaOH and the resultant yellow solution (indicative of dimers) was boiled until colourless. Upon cooling, the pH was re-measured and adjusted if needed. This was repeated until the solution remained colourless.

Minimum Inhibitory Concentration (MIC)

The Minimum Inhibitory Concentration of sodium orthovanadate was determined according to the Clinical and Laboratory Standards Institute (CLSI) guidelines [24]. Briefly, 100 µL of sodium orthovanadate was added to a 96 well plate and serially diluted two-fold in Mueller Hinton broth. An overnight culture of *S.* aureus NCTC 10788 was sub-cultured in fresh Mueller Hinton broth to attain a concentration of 10^6 CFU/mL, and 100 µL of the suspension was added to all relevant wells in the 96 well plate. Bacteria only and broth only controls was carried out in tandem. The plate was incubated statically for 18 h at 32 °C. The MIC was defined as the concentration of sodium orthovanadate that resulted in no detectable bacterial growth, assessed by measuring the optical density at 600 nm.

Minimum Biofilm Inhibitory Concentration (MBIC)

One hundred microliter aliquots of sodium orthovanadate (50 mM in dH_2O, pH 9.2) were added to a 96 well microtiter plate and serially diluted two-fold in Müeller Hinton broth. Next, 100 µL of *S.* aureus NCTC 10788 (10^6 CFU/mL in Mueller Hinton broth) was added to all relevant wells. The microtiter plate was then incubated at 32 °C for 18 h. After incubation, planktonic bacteria were discarded, and the remaining biofilm was washed three times with sterile 50 mM Tris HCl (pH 9.2) and left to dry for 20 min at room temperature. After, 220 µL of 0.1% Crystal Violet (CV) was added to all relevant wells, and the microtiter plate was incubated at room temperature for 30 min. After, the stain was removed, and the plate washed a further three times with sterile 50 mM Tris HCl (pH 9.2) before being left to dry for 3 h at room temperature. Next, 220 µL of 33 % acetic acid was added to the CV-stained biofilms and allowed to incubate for 15 minutes at room temperature, before 100 µL was transferred to a new microtiter plate and the optical density read at 590 nm. The MBIC was defined as the concentration which resulted in no detectable biofilm biomass.

Inhibition of ALP Activity

Overnight cultures of *S.* aureus NCTC 10788 were sub-cultured into fresh Mueller Hinton broth (10^6 CFU/mL) and placed into a 96-well microtiter plate before being incubated at 32 °C for 24 h. After incubation, planktonic bacteria were discarded, and the remaining biofilm was washed three times with sterile 50 mM Tris HCl (pH 9.2) and left to dry for 20 min at room temperature. Next, wells were pre-

treated with various concentrations of sodium orthovanadate (0 – 3.75 mM; pH 9.2) for 30 min at room temperature, before addition of 10 µM **TCF-ALP** in 50 mM Tris-HCl (pH 9.2). Bacterial biofilms were then protected from light and incubated at 32 °C for a further 24 h. From each well, a 200 µL aliquot was removed and centrifuged at 10 000 g for 3 min. The supernatant was subsequently placed into a black 96-well microtiter plate for fluorescence analysis of ALP production.

Colony Biofilm Model

Colony biofilms were prepared as outlined in *Thet et al.* [25] with some modifications. First, 19 mm polycarbonate membranes were UV sterilised for 10 min on Mueller Hinton Agar, before being inoculated with 30 µL Artificial Wound Fluid (AWF; 50% fetal bovine serum in 50% peptone water [0.9% sodium chloride in 0.1% peptone]). Once dry, 50 µL of sub-cultured *S. aureus* NCTC 10788 (10^6 CFU/mL in 50 mM Tris HCl pH 9.2) was placed on the membrane and allowed to dry at room temperature. The inoculated polycarbonate membranes were then incubated for 24 h at 32 °C. Next, biofilms were removed from the agar plate, and placed into 2 mL of 10 µM **TCF-ALP** in 50 mM Tris-HCl (pH 9.2), before being protected from light and incubated at 32 °C for 24 h. After, 1 mL of the suspension was removed and centrifuged at 10 000 g for 3 min. The supernatant was subsequently placed into a black 96-well microtiter plate for fluorescence analysis of ALP production. For **TCF-ALP** hydrogel analysis, biofilms were transferred to a 12-well microtiter plate containing bacteriological agar (to prevent the drying out of the biofilm). A **TCF-ALP-**based PVA hydrogel was subsequently placed on top of the biofilm. After being protected from light and incubated for 24 h at 32 °C, the hydrogels were removed from the biofilm and directly measured for fluorescence intensity.

To determine bacterial concentration, 24 h old biofilms were placed into 10 mL of PBS (pH 7.40) and stripped by sonication (44 KHz) for 15 min twice, with a 60 s interval of vortexing. Viable cells were quantified as outlined **1.5**.

Sterilisation of *Ex-vivo* Porcine Skin Model

Porcine skin was washed with H_2O before being cut into 2 x 2 cm squares, and subsequently underwent three 15-minute vortex cycles of washing in sterile dH_2O. After, porcine skin was vortexed once for 15 min in 70 % ethanol, before a further two washes with sterile dH_2O. Finally, the skin was UV-irradiated using a commercial UV source (Hamanatsu, Japan) equipped with a 254 nm UV lamp for 10 min before use.

ALP Activity

A 10 µL aliquot of *S. aureus* NCTC 10788 (10^8 CFU/mL in 50 mM Tris HCl pH 9.2) was added to the sterilised skin and left to dry for 20 min at room temperature. Next, 1 mL of 10 µM **TCF-ALP** in 50

mM Tris-HCl (pH 9.2) was added to the skin via use of a Franz Cell. Inoculated porcine skin was then protected from light and incubated at 32 °C for 24 h. After, the suspension was removed and centrifuged at 10 000 g for 3 min. The supernatant was subsequently placed into a black 96-well microtiter plate for fluorescence analysis of ALP production. For **TCF-ALP** hydrogel analysis, a 100 µM **TCF-ALP** based PVA hydrogel was subsequently placed on the top of the inoculated skin. After being protected from light and incubated for 24 h at 32 °C, the hydrogels were removed from the skin and directly measured for fluorescence intensity.

Fluorescence imaging of *Staphylococcus aureus* using Confocal Laser-Scanning Microscope (CLSM)

Overnight cultures of Methicillin-resistant *S. aureus* (MRSA) ATCC 43300 were grown in Luria Bertani (LB) broth at 37°C for 14-18 h. After reaching an optical density of 600 nm of ~ 1 (c. 5×10^8 CFU/mL). The obtained bacteria fluid was then diluted to ten times by water, underwent centrifugation (8000 rpm, 5 min) and re-suspended in an equal volume of **TCF-ALP** (40 µM) in water. For ALP-inhibiting assay, phosphatase inhibitor cocktail A (Beyotime, containing 250 mM sodium fluoride, 50 mM sodium pyrophosphate, 50 mM β-glycerophosphate and 50 mM sodium orthovanadate) was used to inhibit bacterial phosphatase. The diluted bacteria fluid was pre-treated with two different concentrations of phosphatase inhibitor cocktail A (1% and 2%) respectively and incubated for 1 h. Then, samples underwent centrifugation (8000 rpm, 5 min) before re-suspended in an equal volume of **TCF-ALP** (40 µM) in water. After treating **TCF-ALP**, all suspensions were further incubated at room temperature in the dark for at least 12 h, until a colour change was observed in the non-inhibitor group. After incubation, all suspensions were centrifuged at 8000 rpm for 5 min to obtain bacteria precipitates, before washing for at least three times with PBS buffer. The clean bacteria precipitate of each group was then suspended in pure water, placed (100 µL each) onto one microscope slice respectively and dried at room temperature. CLSM images were obtained using a Nikon AIR Confocal Laser-Scanning Microscope equipped with a 60 x oil-immersion objective lens. The excitation and emission wavelengths were 561 nm and 606 nm, respectively. The fluorescence intensities of the CLSM images were measured by Image-J.

2. Role of Bacterial Growth Media on ALP Production

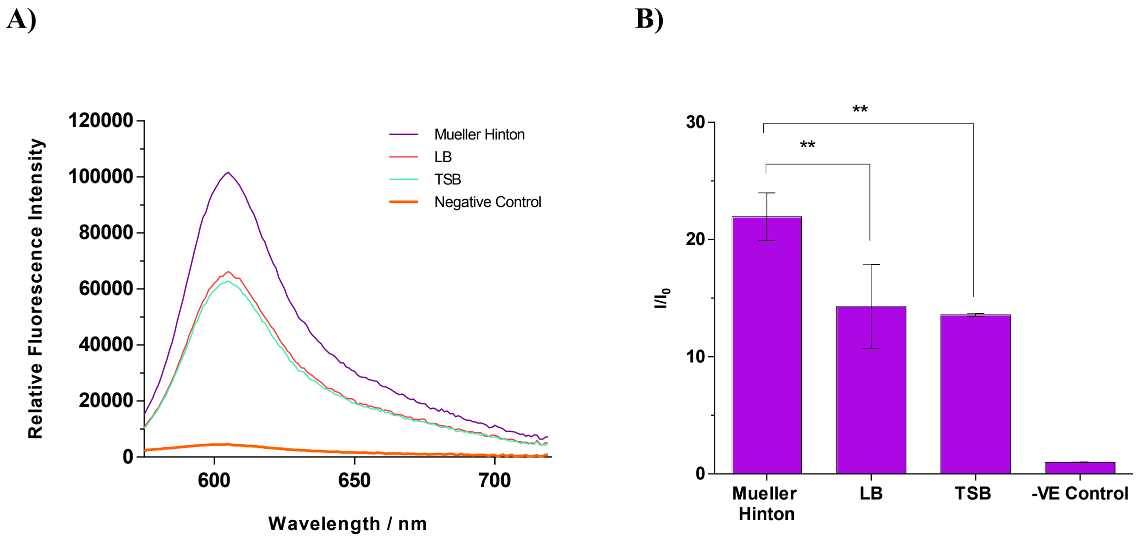

Figure S1: *Staphylococcus aureus* NCTC 10788 (10^8 CFU/mL) was grown in: Mueller Hinton, LB or TSB before being inoculated for 1 h with **TCF-ALP** (10 μM) in 50 mM Tris-HCl buffer pH = 9.2 at 32 °C. **A)** Fluorescence spectra and **B)** corresponding change in fluorescence (I/I_0). λ_{ex} = 542 (bandwidth 15) nm. λ_{em} = 606 nm. Error bars indicate standard deviation (n = 3). Statistical significance was assessed by performing a One-way ANOVA followed by Turkey post-hoc test. *p* values are indicated (**, 0.01).

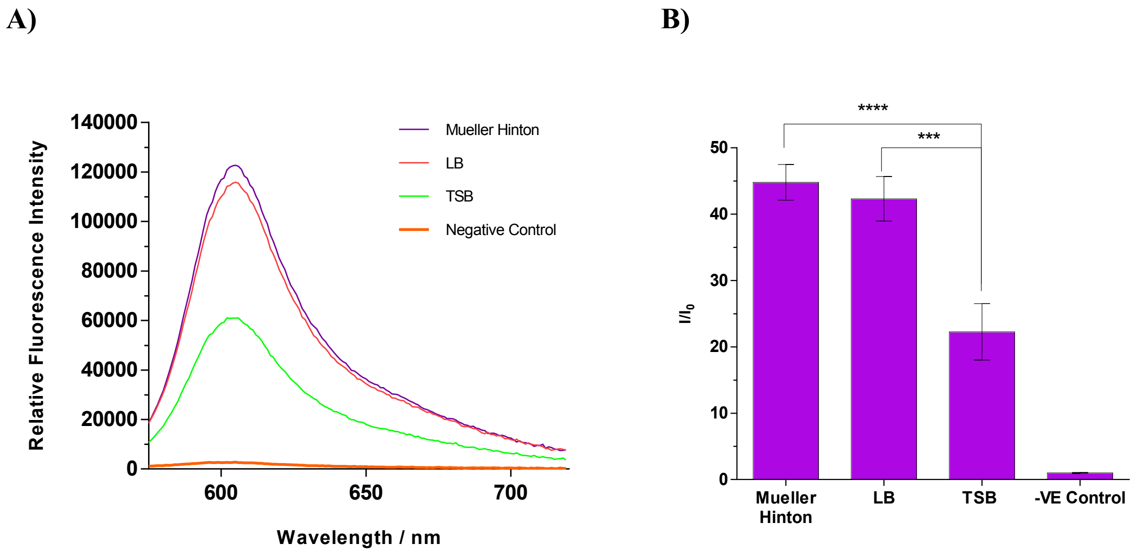

Figure S2: *Staphylococcus aureus* NCTC 10788 (10^8 CFU/mL) was grown in either Mueller Hinton, LB or TSB before being inoculated for 24 h with **TCF-ALP** (10 μM) in 50 mM Tris-HCl buffer pH = 9.2 at 32 °C. **A)** Fluorescence spectra and **B)** corresponding change in fluorescence (I/I_0). λ_{ex} = 542 (bandwidth 15) nm. λ_{em} = 606 nm. Error bars indicate standard deviation (n = 3). Statistical significance was assessed by performing a One-way ANOVA followed by Turkey post-hoc test. *P* values are indicated (****, < 0.0001; ***, 0.001).

3. Washing Steps

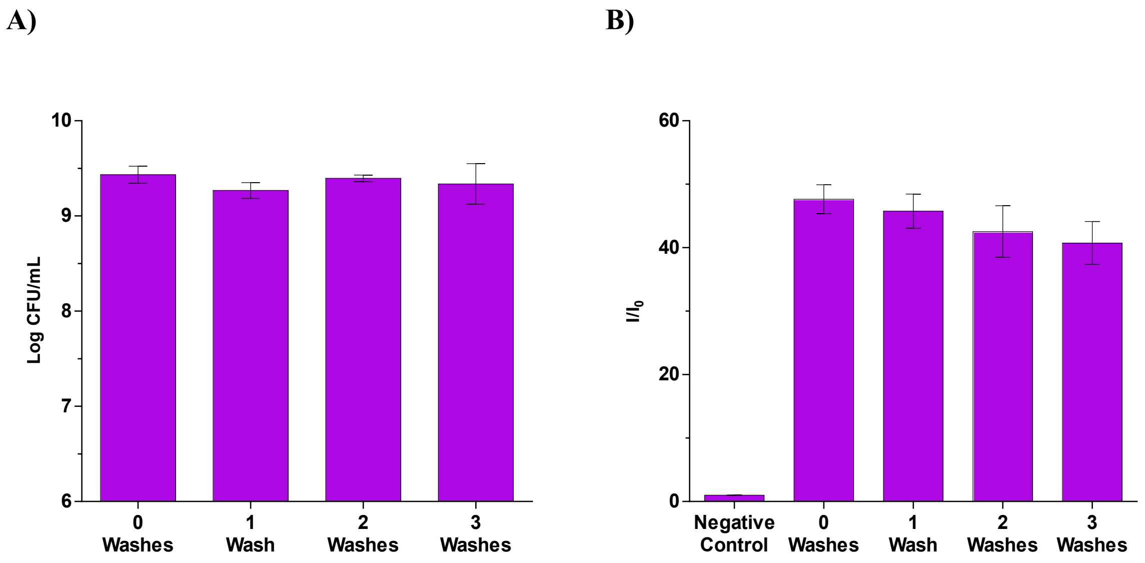

Figure S3: **A)** Log CFU/mL of *Staphylococcus aureus* NCTC 10788 after increasing wash cycles (0 – 3) and **B)** Change in fluorescence (I/I_0) of **TCF-ALP** (10 µM) after 24 h incubation with washed suspensions of *Staphylococcus aureus* NCTC 10788 (10^8 CFU/mL) in 50 mM Tris-HCl buffer pH = 9.2 at 32 °C. λ_{ex} = 542 (bandwidth 15) nm. λ_{em} = 606 nm. Statistical significance was assessed by performing a One-way ANOVA followed by Turkey post-hoc test.

4. Incubation Temperatures

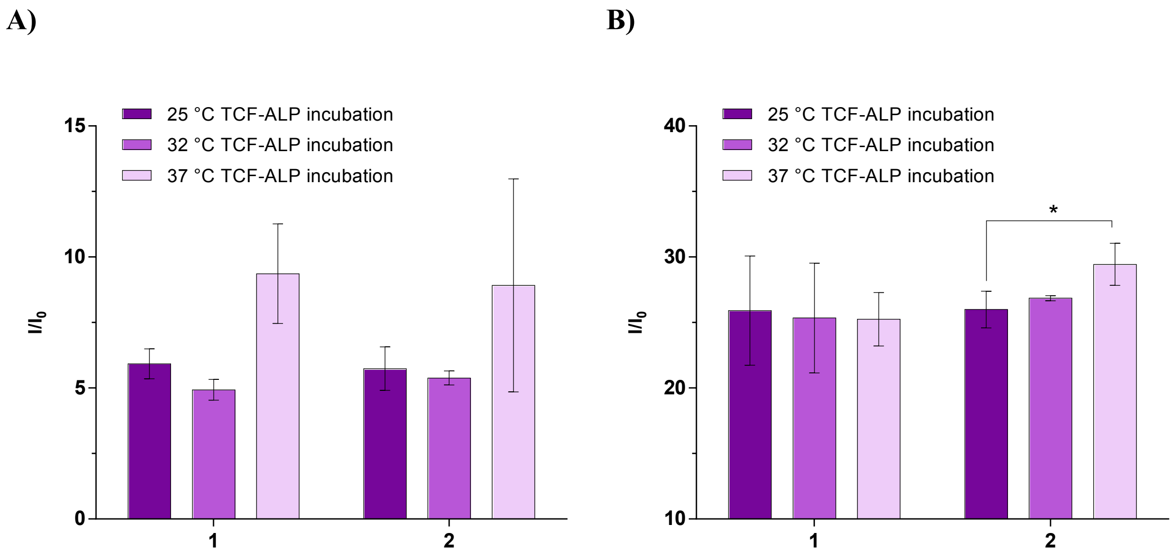

Figure S4: Change in fluorescence (I/I_0) of **TCF-ALP** (10 µM) after **A)** 1 h and **B)** 24 h incubation with *Staphylococcus aureus* NCTC 10788 (10^8 CFU/mL) in 50 mM Tris-HCl buffer pH = 9.2. Bacterial cultures were grown at either 32 °C (Group **1**) or 37 °C (Group **2**) and incubated with **TCF-ALP** (10 µM) at 25 °C, 32 °C or 37 °C. λ_{ex} = 542 (bandwidth 15) nm. λ_{em} = 606 nm. Statistical significance was assessed by performing a t-test with Welch's correction. *p* values are indicated (*, 0.0499).

5. Limit of Detection

Fluorescence intensity were measured for bacterial concentrations ranging from 10^5 – 10^{10} CFU/mL. The fluorescence intensities were normalised and plotted against a Log CFU/mL scale. The linear portion of the graph was obtained, and the Limit of Detection (LOD) was defined as the x-intercept.

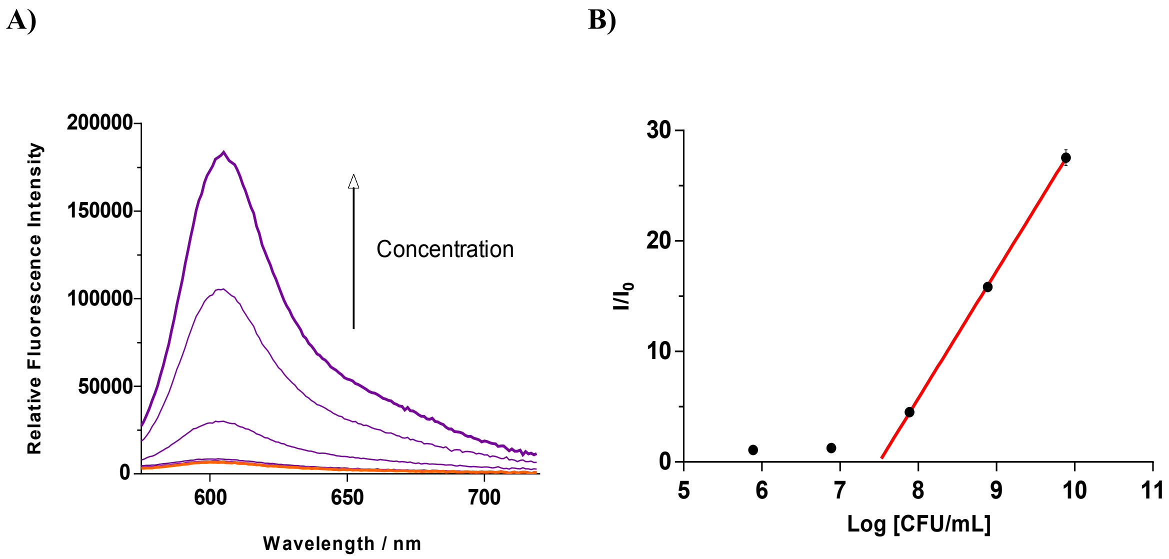

Figure S5: **A)** Fluorescence spectra of **TCF-ALP** (10 μM) after 1 h incubation with various concentrations of *Staphylococcus aureus* NCTC 10788 (0 – 10^9 CFU/mL) in 50 mM Tris-HCl buffer pH = 9.2 at 32 °C, and **B)** corresponding selectivity bar chart. λ_{ex} = 542 (bandwidth 15) nm. λ_{em} = 606 nm. (X-intercept 7.501 = 3.17 x 10^7 CFU/mL; Y = 11.52X – 86.38; R^2 0.9983).

Table S2: Table showing Limits of Detection (LOD) for fluorescent and colorimetric probes for the detection of bacterial enzymes.

Bacteria	Enzyme	Detection Method	LOD (CFU/mL)	Time	Year	Ref.
Salmonella	C8-Esterase	Chemi-luminescence	10 cells	6 h	2019	[26]
Listeria monocytes	PI-PLC	Chemi-luminescence	10^4	24 h	2019	[26]
Pseudomonas aeruginosa	Carbapenemase	Chemi-luminescence	10^7	20 min	2020	[27]
Klebsiella pneumonia	Carbapenemase	Chemi-luminescence	10^7	20 min	2020	[27]
Escherichia coli	β-lactamase	Chemi-luminescence	10^5	30 min	2020	[28]
Escherichia coli	β-lactamase	Fluorescence	10^7	30 min	2020	[28]
Escherichia coli	β -galactosidase	Colorimetric	10^5 with cell lysis, 10^7 without.	40 min	2017	[29]
Escherichia coli	β -galactosidase	Colorimetric hydrogel	10^2	60 min	2010	[30]
Escherichia coli	β-glucuronidase	Fluorescence*	10^2	30 min	2010	[31]
Escherichia coli	β-glucuronidase	Fluorescence	10^5	-	1999	[32]
Escherichia coli	Numerous	Colorimetric	10^1 with 7 h enrichment, 10^7 without	4 h for enrichment, 60 min without	2019	[33]

*this work developed a novel hand-held fluorimeter to detect *Escherichia coli*.

6. Fluorescence imaging of *Staphylococcus aureus* using Confocal Laser-Scanning Microscope (CLSM)

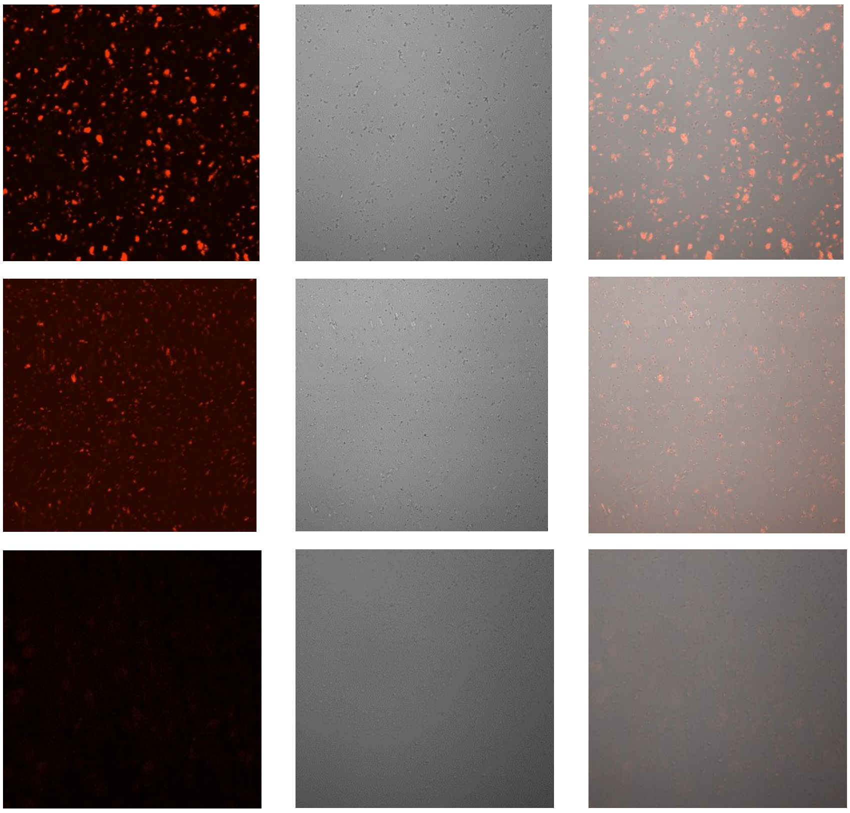

Figure S6: CLSM images of *S. aureus* ATCC 4300 after a minimum of 12 h incubation with **TCF-ALP** (40 μM) with and without the presence of an inhibitor cocktail (Beyotime, containing 250 mM sodium fluoride, 50 mM sodium pyrophosphate, 50 mM β-glycerophosphate and 50 mM sodium orthovanadate) at 25 °C. CLSM images were obtained using a Nikon AIR Confocal Laser-Scanning Microscope equipped with a 60x oil-immersion objective lens. λ_{ex} = 561 nm, λ_{em} = 606 nm, n = 3.

A)

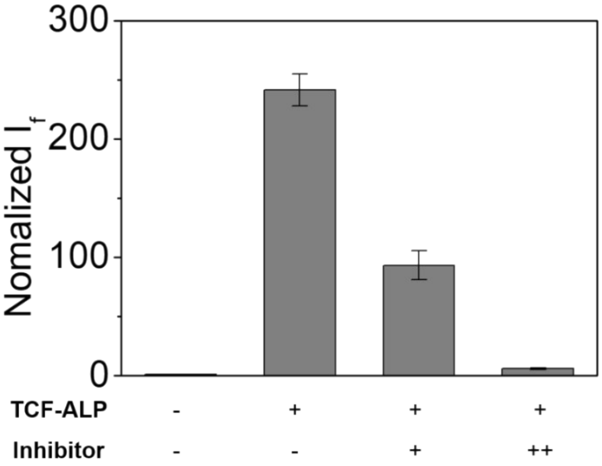

B)

0 h **12 h**

S. aureus ATCC 43300 and **TCF-ALP**

S. aureus ATCC 43300, **TCF-ALP** and 2 µL inhibitor cocktail

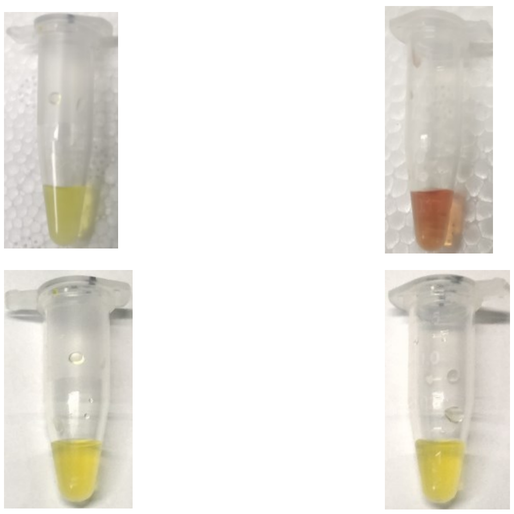

Figure S7: A) Fluorescence intensities of the CLSM images of *S. aureus* ATCC 4300 after a minimum of 12 h incubation with **TCF-ALP** with and without the presence of an inhibitor cocktail (Beyotime, containing 250 mM sodium fluoride, 50 mM sodium pyrophosphate, 50 mM β-glycerophosphate and 50 mM sodium orthovanadate) at 25 °C. The fluorescence intensities of the CLSM images were measured by Image-J, n = 3, error bars indicate standard deviation. **B)** Images of *S. aureus* ATCC 4300 after 12 h incubation with **TCF-ALP** with or without the presence of an inhibitor cocktail (Beyotime, containing 250 mM sodium fluoride, 50 mM sodium pyrophosphate, 50 mM β-glycerophosphate and 50 mM sodium orthovanadate) at 25 °C.

7. Additional Selectivity Experiments and Cell Count for Selectivity Assay

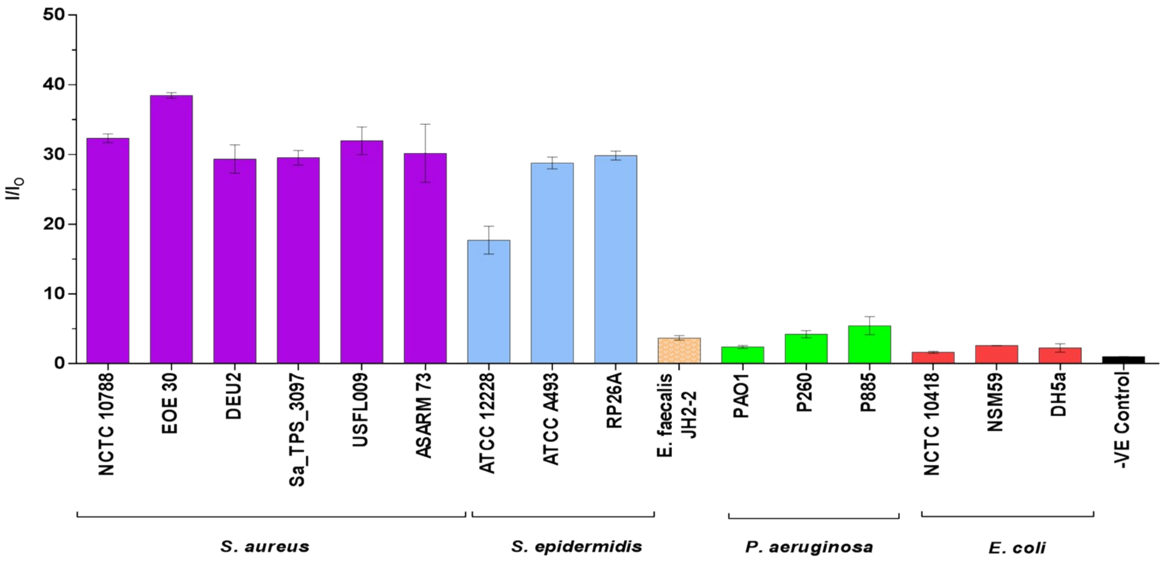

Figure S8: Selectivity bar chart of **TCF-ALP** (10 µM) in 50 mM Tris-HCl buffer pH = 9.2 after 24 h incubation with various bacterial strains (10^8 CFU/mL) at 32 °C. λ_{ex} = 542 (bandwidth 15) nm. λ_{em} = 606 nm. Error bars indicate standard deviation (n = 3).

Cell counts were performed for bacterial strains that were unable to elicit a fluorescence response upon incubation with **TCF-ALP**. **Figure S8** shows that the bacterial concentration of all *E. coli* strains (NCTC 10418, NSM59, and DH5α) were unaffected after 24 h incubation with **TCF-ALP**. All *P. aeruginosa* strains (PAO1, P260, P885) had a statistically significant reduction in bacterial concentration; however, this correlated to a 1-log reduction, which should have a minimal effect on the efficacy of **TCF-ALP**. Therefore, other reasons must explain why *E. coli* and *P. aeruginosa* failed to produce a 'turn on' of **TCF-ALP**.

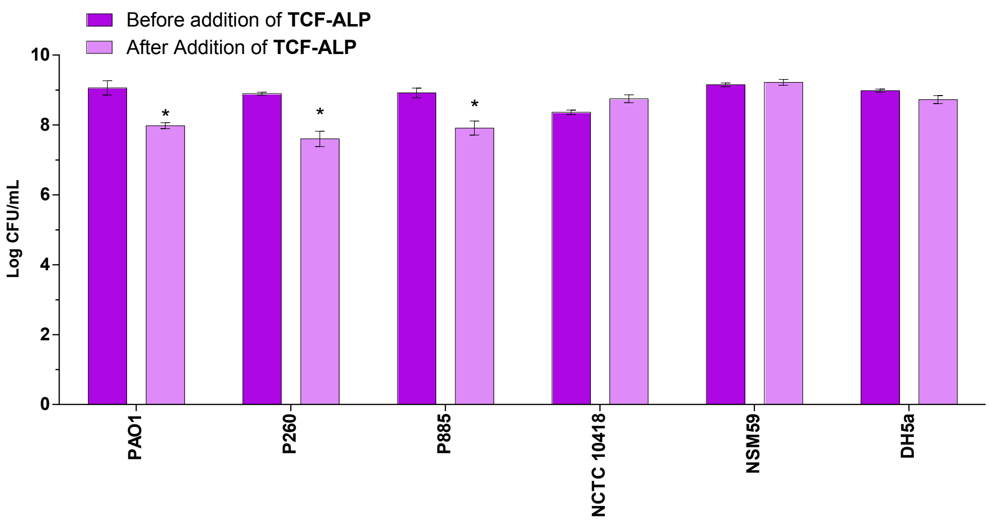

Figure S9: Log CFU/mL of *P. aeruginosa* and *E. coli* strains before and after 24 h incubation with 10 µM **TCF-ALP** in Tris-HCl, pH = 9.2. Error bars show standard deviation (n = 3). Statistical significance was assessed by performing a t-test. *p* values are indicated (*, < 0.05).

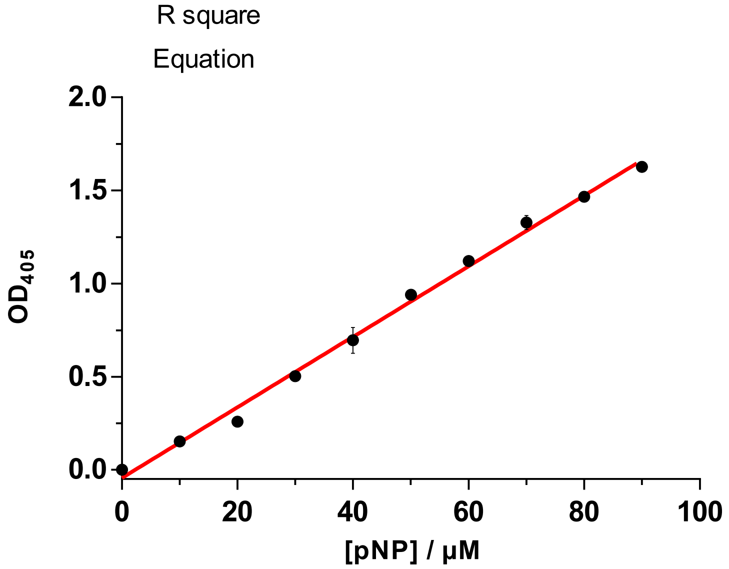

Figure S10: Calibration curve for *p*-nitrophenol (*p*-NP) in 50 mM Tris-HCl containing 250 mM NaOH at 25°C. Error bars indicate standard deviation (n=3).

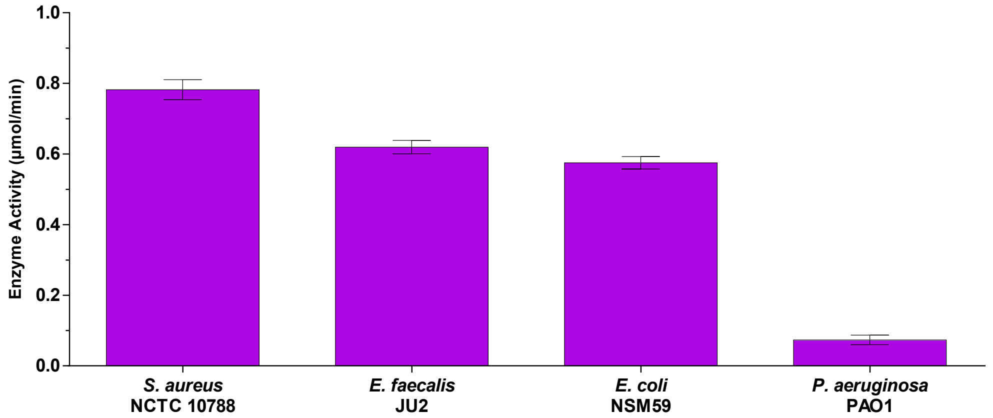

Figure S11: ALP activity (µmol/min) of bacterial strains tested using *p*-nitrophenyl phosphate in 50 mM Tris-HCl (pH 9.2). Experiments were conducted at 32 °C and error bars indicate standard deviation (n=3). 1 U (µmol/min) is defined as the amount of the enzyme that catalyzes the conversion of one micromole of substrate per minute under the specified conditions of the assay method.

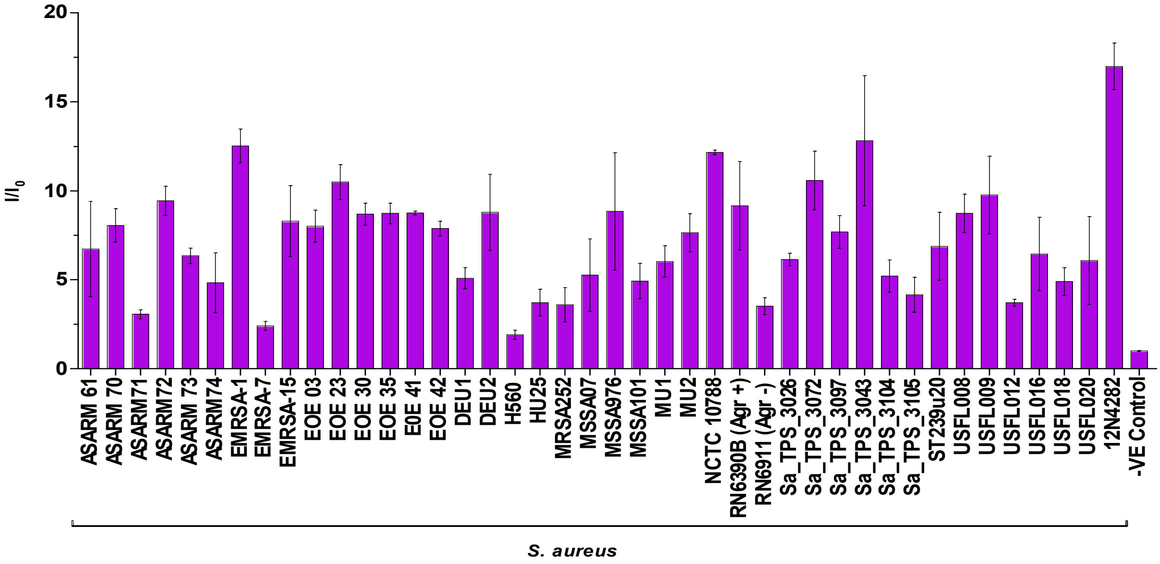

Figure S12: Selectivity bar chart of **TCF-ALP** (10 µM) in 50 mM Tris-HCl buffer pH 9.2 after 1 h incubation with various bacterial strains (10^8 CFU/mL) at 32 °C. λ_{ex} = 542 (bandwidth 15) nm. λ_{em} = 606 nm. Error bars indicate standard deviation (n = 3).

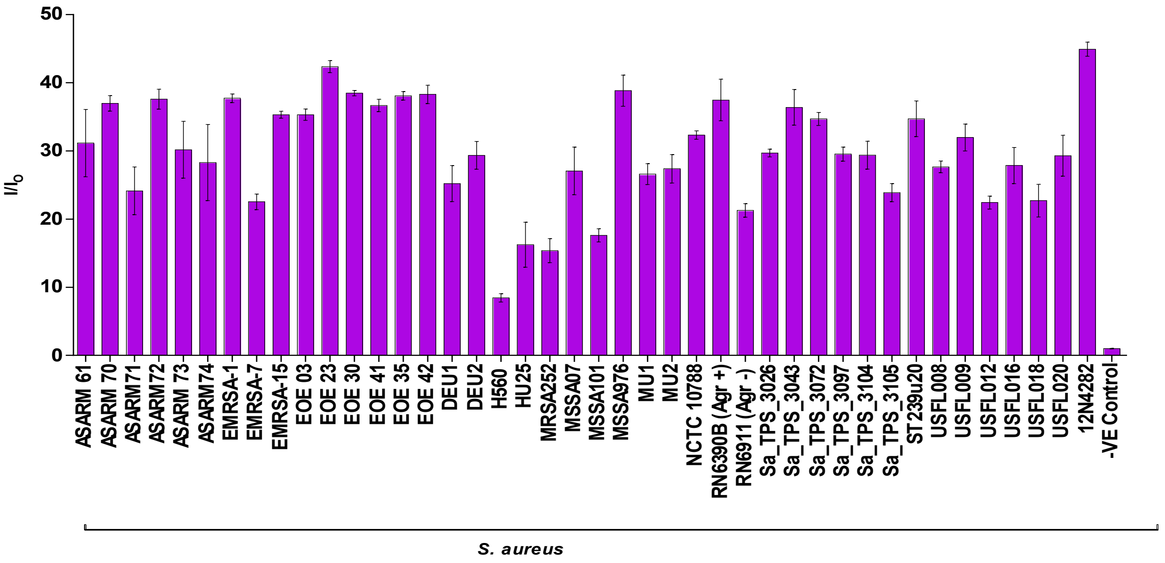

Figure S13: Selectivity bar chart of **TCF-ALP** (10 µM) in 50 mM Tris-HCl buffer pH 9.2 after 24 h incubation with various bacterial strains (10^8 CFU/mL) at 32 °C. λ_{ex} = 542 (bandwidth 15) nm. λ_{em} = 606 nm. Error bars indicate standard deviation (n = 3).

8. 96-well Microtiter Plate Biofilms

A) **B)**

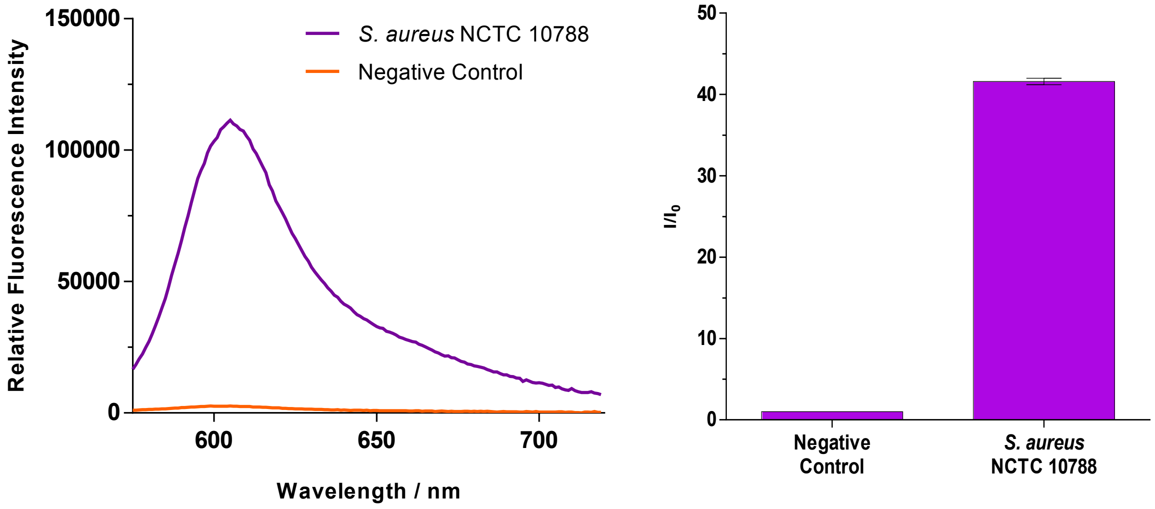

Figure S14: **A)** Fluorescence spectra of **TCF-ALP** (10 µM) after 24 h incubation with a 96-well plate biofilm of *S. aureus* NCTC 10788 in 50 mM Tris-HCl, buffer, pH = 9.2 at 32 °C**,** and **B)** corresponding selectivity bar chart. λ_{ex} = 542 (bandwidth 15) nm. λ_{em} = 606 nm. Error bars indicate standard deviation (n = 3).

9. Sodium Orthovanadate Minimum Inhibitory Concentration and Minimum Biofilm Inhibition Concentration

A) B)

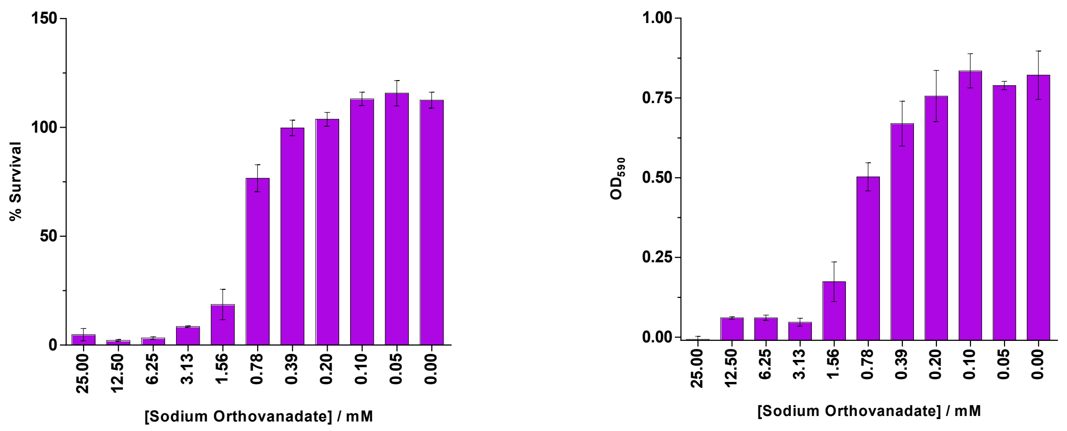

Figure S15: **A)** Minimum Inhibitory Concentration (MIC) of sodium orthovanadate for *Staphylococcus aureus* NCTC 10788. MIC was found to be 6.25 - 3.13 mM. **B)** Minimum Biofilm Inhibitory Concentration (MBIC) of sodium orthovanadate for *Staphylococcus aureus* NCTC 10788. Biofilm biomass was quantified using crystal violet biofilm staining by measuring the absorbance at 590 nm MBIC was found to be 3.13 – 1.56 mM. Error bars show standard deviation (n = 3).

10. Effect of Sodium Orthovanadate on ALP Production in *S. aureus* Biofilms

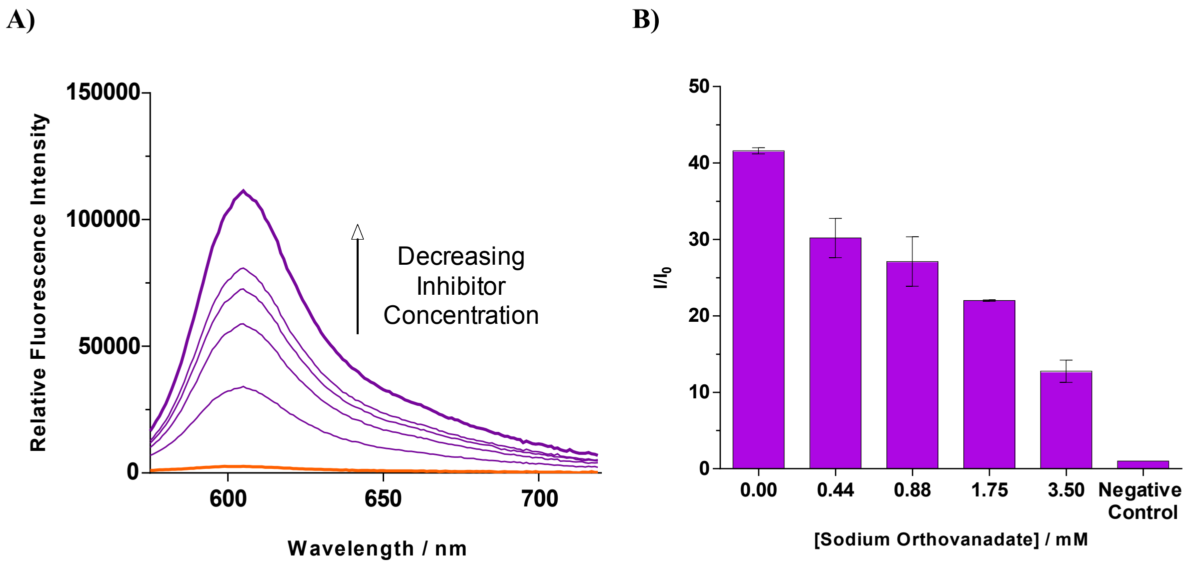

Figure S16: **A)** Fluorescence spectra of **TCF-ALP** (10 µM) in 50 mM Tris-HCl buffer, pH = 9.2 after 24 h at 32 °C incubation with *S. aureus* NCTC 10788 (10^8 CFU/mL) pre-treated with various concentrations of sodium orthovanadate (0-3.50 mM) for 30 min prior to testing, and **B)** corresponding selectivity bar chart. λ_{ex} = 542 (bandwidth 15) nm. λ_{em} = 606 nm. Error bars indicate standard deviation (n = 3).

11. Colony Biofilm Models

A) B)

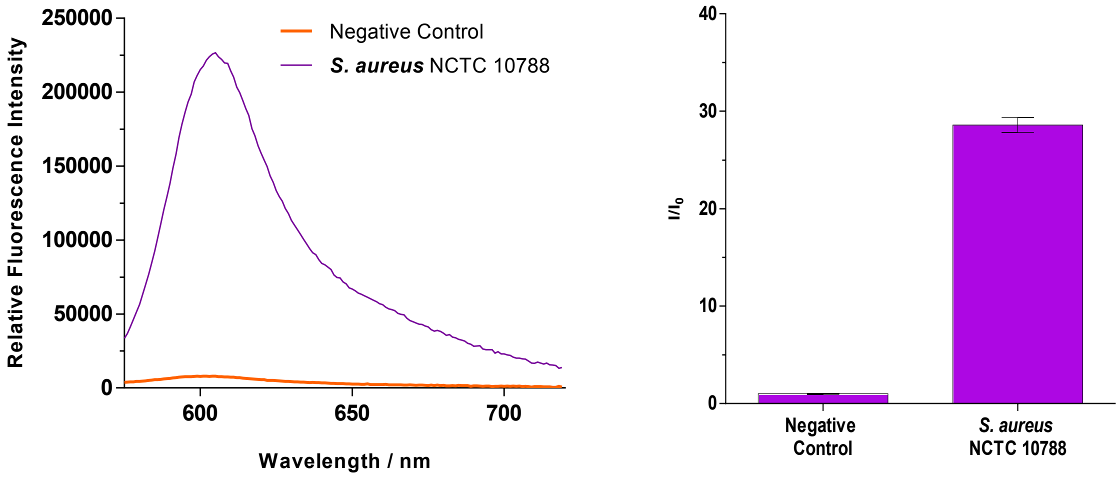

Figure S17: **A)** Fluorescence spectra of **TCF-ALP** (10 µM) after 1 h incubation with biofilms of *S. aureus* NCTC 10788 (10^{11} CFU/membrane) in 50 mM Tris-HCl buffer, pH = 9.2 at 32 °C and **B)** corresponding selectivity bar chart. λ_{ex} = 542 (bandwidth 15) nm. λ_{em} = 606 nm. Error bars indicate standard deviation (n = 3).

A) B)

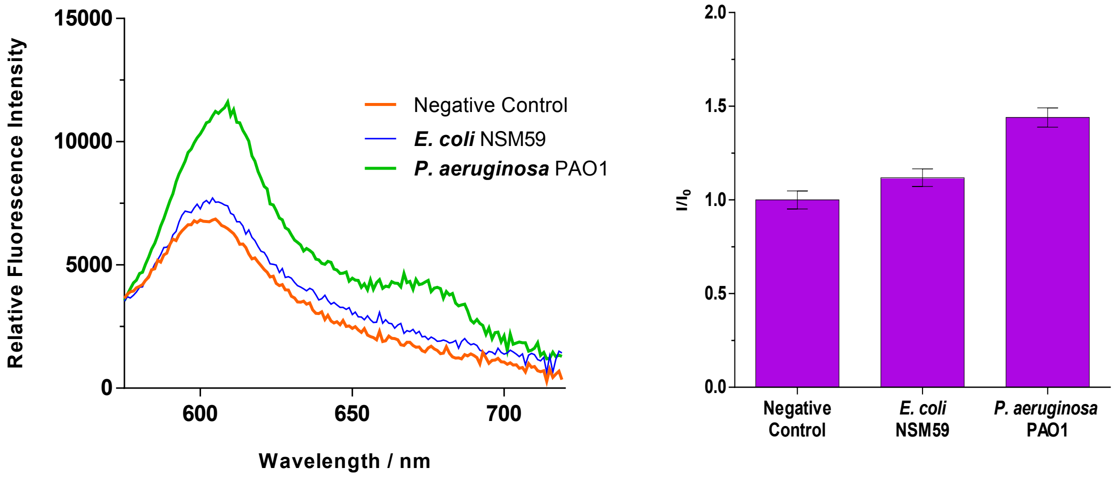

Figure S18: A) Fluorescence spectra of **TCF-ALP** (10 µM) after 1 h incubation with biofilms of *E. coli* NSM59 and *P. aeruginosa* PAO1 (10^{11} CFU/membrane) in 50 mM Tris-HCl buffer, pH = 9.2 at 32 °C and **B)** corresponding selectivity bar chart. λ_{ex} = 542 (bandwidth 15) nm. λ_{em} = 606 nm. Error bars indicate standard deviation (n = 3).

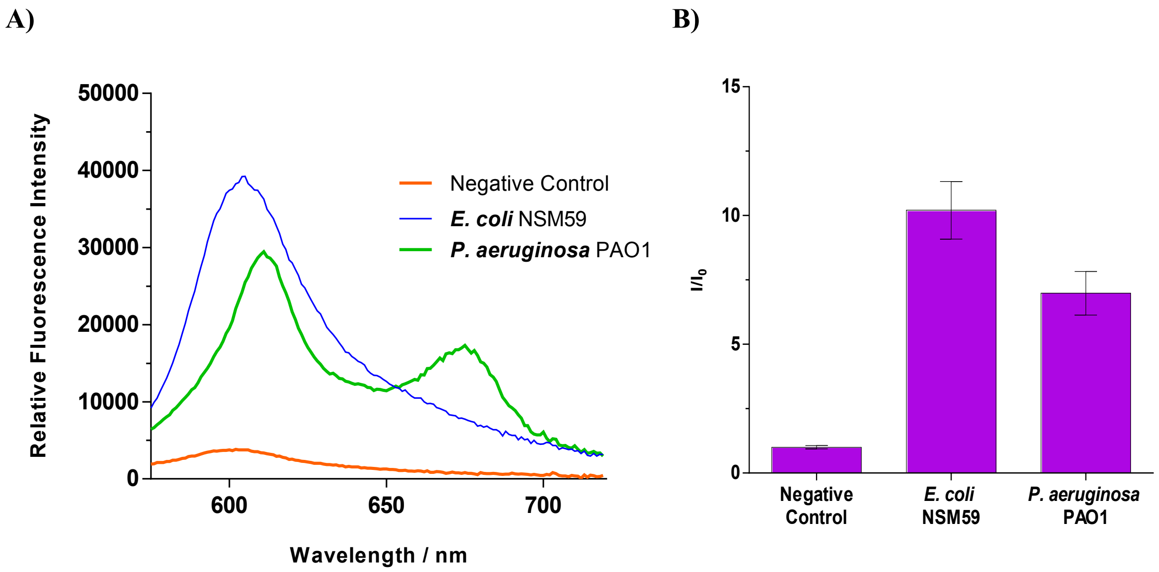

Figure S19: A) Fluorescence spectra of TCF-ALP (10 μM) after 24 h incubation with biofilms of *E. coli* NSM59 and *P. aeruginosa* PAO1 (10^{11} CFU/membrane) in 50 mM Tris-HCl buffer pH = 9.2 at 32 °C and **B)** corresponding selectivity bar chart. λ_{ex} = 542 (bandwidth 15) nm. λ_{em} = 606 nm. Error bars indicate standard deviation (n = 3).

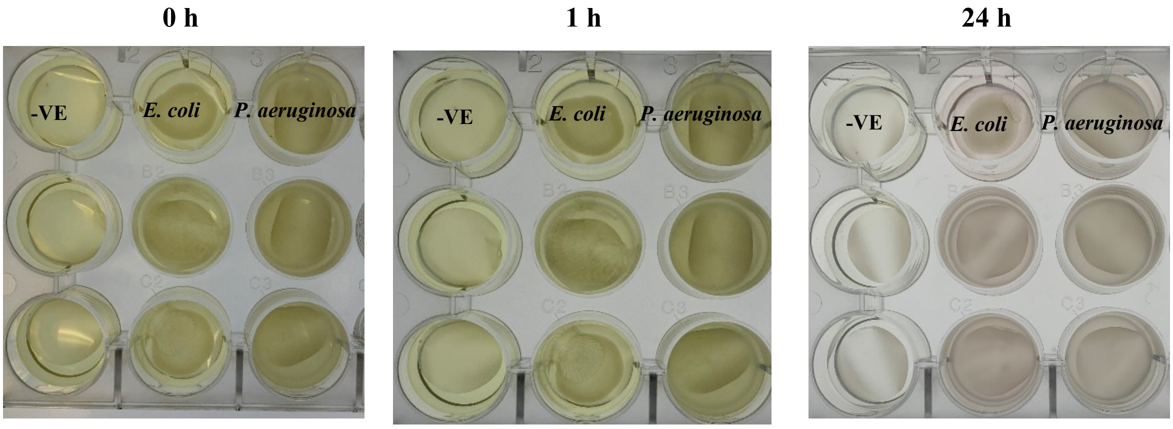

Figure S20: Images taken of negative controls (Membrane and Artificial Wound Fluid (AWF) only) and biofilms of *E. coli* NSM59 and *P. aeruginosa* PAO1 (10^{11} CFU/membrane) after 0, 1, and 24 h incubation with 10 μM **TCF-ALP** in 50 mM Tris-HCl buffer, pH = 9.2 at 32 °C.

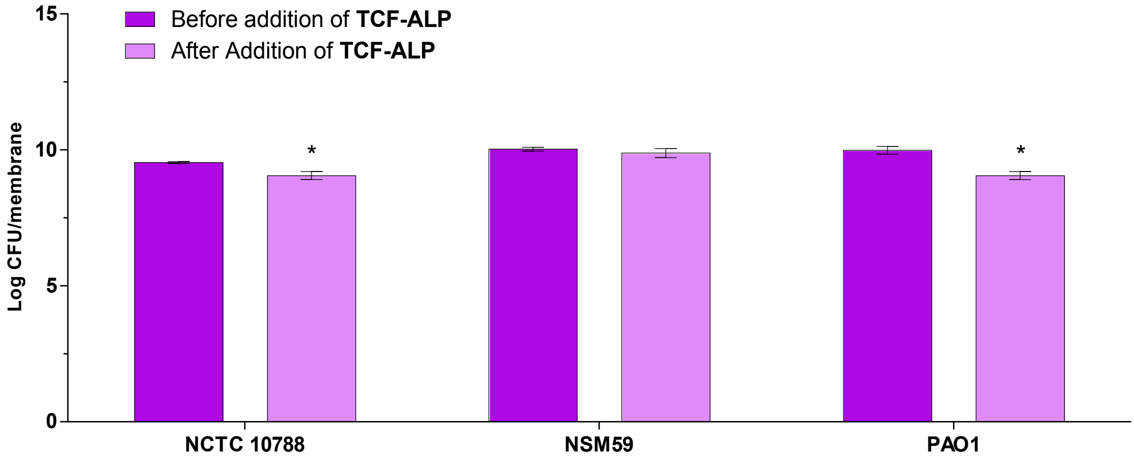

Figure S21: Log CFU/mL of *S. aureus* NCTC 10788, *E. coli* NSM59, and *P. aeruginosa* PAO1 biofilms after 24 h incubation at 32°C with solutions of **TCF-ALP** (10 μM) in Tris-HCl, pH 9.2. Error bars show standard deviation (n = 3). Statistical significance was assessed by performing a t-test. *p* values are indicated (*, < 0.05).

12. TCF-ALP Hydrogels for Planktonic Bacterial Detection

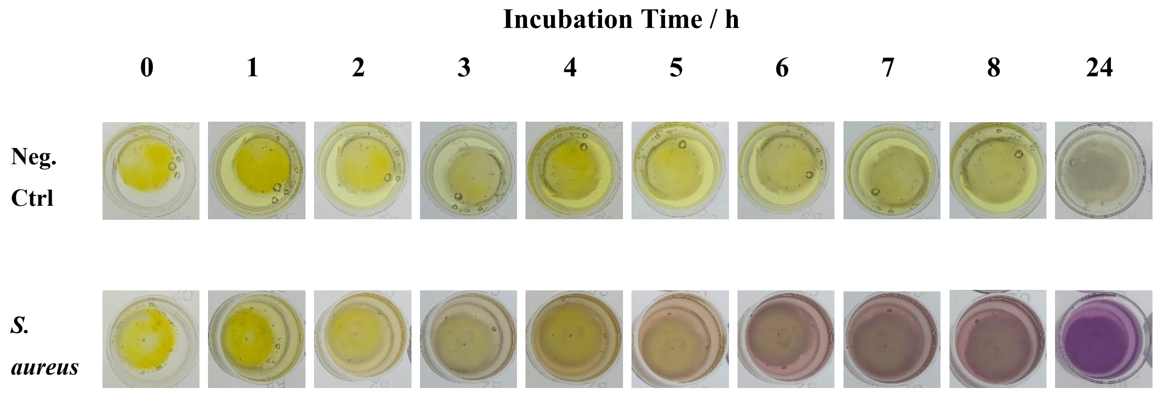

Figure S22: Images taken of 10% w/v PVA hydrogels loaded with 100 μM **TCF-ALP** in 50 mM Tris-HCl buffer, pH = 9.2 after 0 – 8 h and 24 h incubation at 32 °C with *S. aureus* NCTC 10788 planktonic cultures (c. 10^8 CFU/mL).

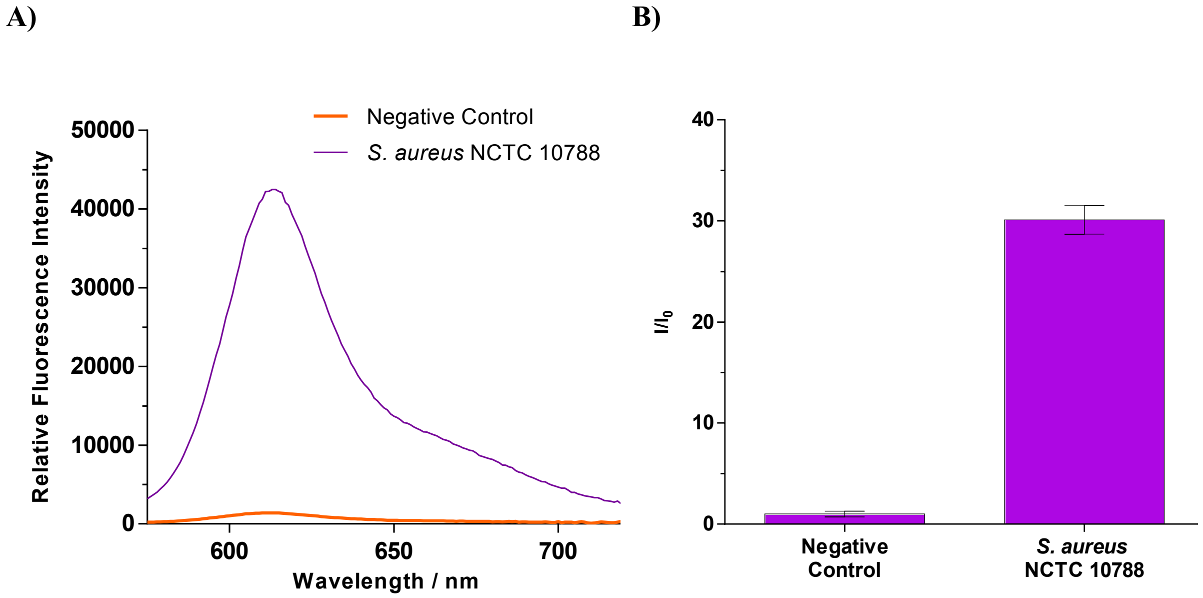

Figure S23: **A)** Fluorescence spectra of **TCF-ALP** (100 μM) in a 10% w/v PVA hydrogel after 24 h incubation at 32 °C with planktonic cultures of *S. aureus* NCTC 10788 (c. 10^8 CFU/mL)**,** and **B)** corresponding selectivity bar chart. λ_{ex} = 542 (bandwidth 15) nm. λ_{em} = 606 nm. Error bars indicate standard deviation (n = 3).

13. TCF-ALP Hydrogels with Colony Biofilm Models of *S. aureus*

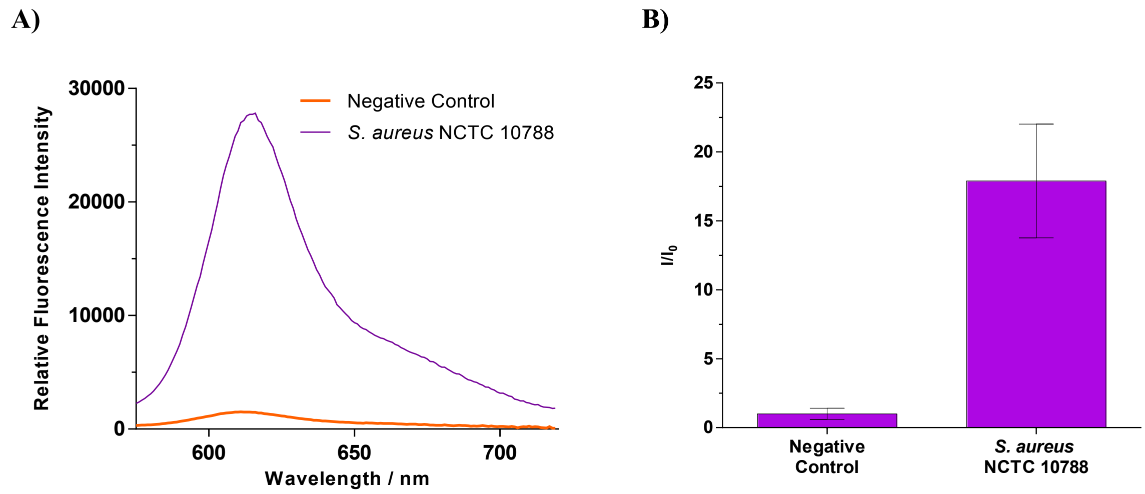

Figure S24: **A)** Fluorescence spectra of **TCF-ALP** (100 µM) in a 10% w/v PVA hydrogels after 24 h incubation at 32 °C with *S. aureus* NCTC 10788 biofilms (10^{11} CFU/membrane)**,** and **B)** corresponding selectivity bar chart. λ_{ex} = 542 (bandwidth 15) nm. λ_{em} = 606 nm. Error bars indicate standard deviation (n = 3).

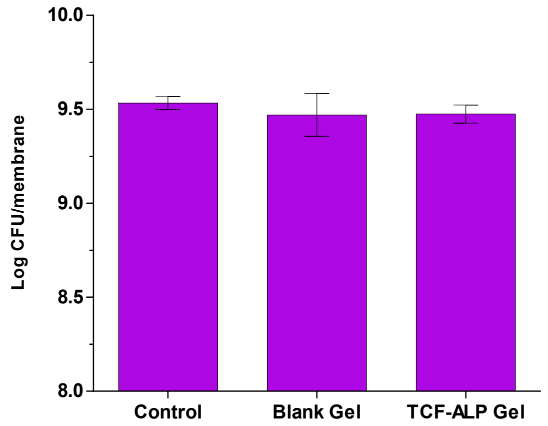

Figure S25: Log CFU/mL of *S. aureus* NCTC 10788 colony biofilm model biofilms after 24 h incubation at 32°C with 10% w/v PVA hydrogels loaded with **TCF-ALP (**100 µM). Error bars show standard deviation (n = 3).

14. *Ex vivo* experiments using TCF-ALP

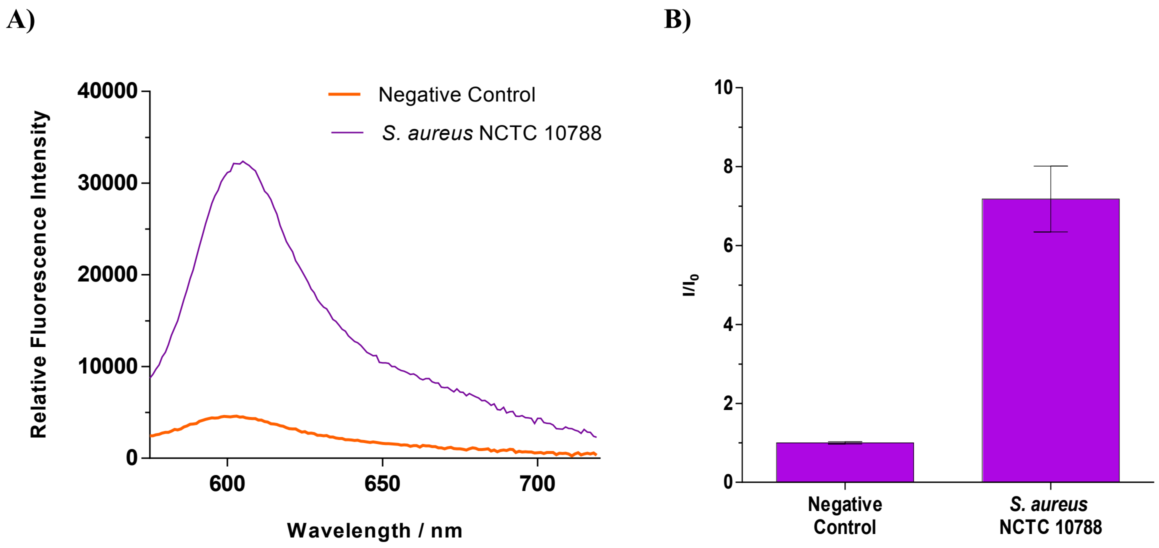

Figure S26: **A)** Fluorescence spectra of **TCF-ALP** (10 μM) in 50 mM Tris-HCl buffer, pH = 9.2 after 24 h incubation with *S. aureus* NCTC 10788 inoculated porcine skin at 32 °C**,** and **B)** corresponding selectivity bar chart. λ_{ex} = 542 (bandwidth 15) nm. λ_{em} = 606 nm. Error bars indicate standard deviation (n = 3).

Negative Control ***S. aureus*** **NCTC 10788**

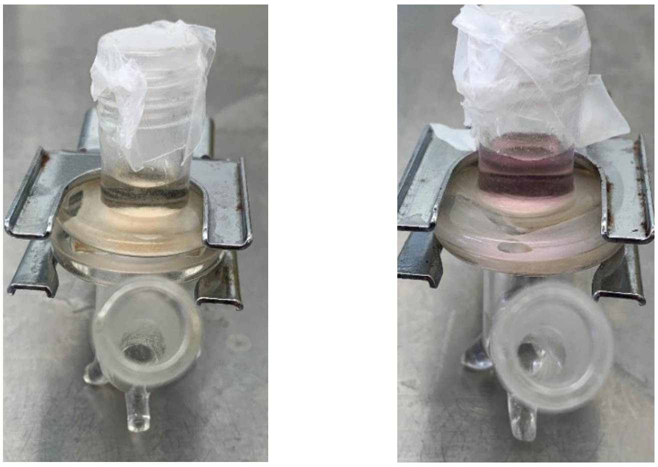

Figure S27 : Images of the negative control (no bacteria) and *S. aureus* NCTC 10788 on porcine skin after 24 h incubation at 32 °C with 10 μM **TCF-ALP** in 50 mM Tris-HCl buffer, pH = 9.2.

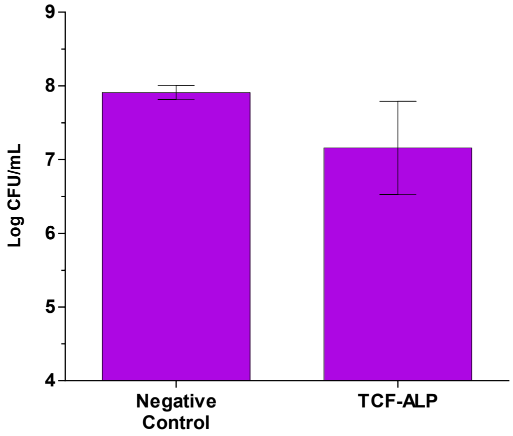

Figure S28: Log CFU/mL of *S. aureus* NCTC 10788 incubated on porcine skin for 24 h at 32°C with a solution of **TCF-ALP** in 50 mM Tris-HCl, pH 9.2 (10 µM; 1 mL). The negative control was undertaken using a solution of 50 mM Tris-HCl, pH 9.2 (1 mL). Error bars show standard deviation (n = 3).

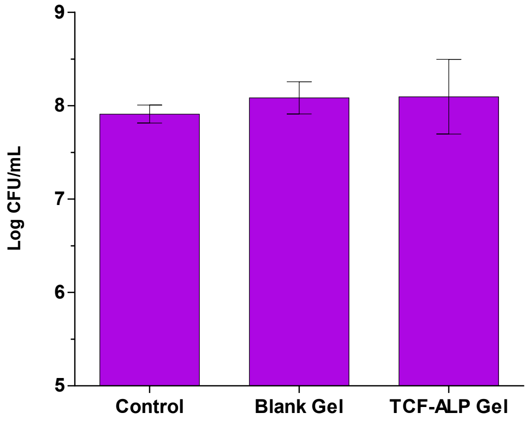

Figure S29: Log CFU/mL of *S. aureus* NCTC 10788 on porcine skin after 24 h incubation at 32°C with 10% w/v PVA hydrogels loaded with **TCF-ALP (**100 µM). Error bars show standard deviation (n = 3).

15. References

1. L. Gwynne, A. C. Sedgwick, J. E. Gardiner, G. T. Williams, G. Kim, J. P. Lowe, J.-Y. Maillard, A. T. A. Jenkins, S. D. Bull, J. L. Sessler, J. Yoon and T. D. James. *Front. Chem.*, 2019, **7**, 255.
2. Culture collections: Staphylococcus aureus. https://www.phe-culturecollections.org.uk/products/bacteria/detail.jsp?refId=NCTC%2010788&collection=nctc. Date accessed: 02/10/2020.
3. C. Shaw, J. M. Stitt and S. T. Cowan. J. Gen. Microbiol. ,1951, **5**, 1010-1023.
4. M. Recker, M. Laabei, M. S. Toleman, S. Reuter, R. B. Saunderson, B. Blane, M. E. Török, K. Ouadi, E. Stevens, M. Yokoyama, J. Steventon, L. Thompson, G. Milne, S. Bayliss, L. Bacon, S. J. Peacock and R. C. Massey. Nat. Microbiol., 2017, **2**, 1381-1388
5. M. Laabei, M. Recker, J. K. Rudkin, M. Aldeljawi, Z. Gulay, T. J. Sloan, P. Williams, J. L. Endres, K. W. Bayles, P. D. Fey, V. K. Yajjala, T. Widhelm, E. Hawkins, K. Lewis, S. Parfett, L. Scowen, S. J. Peacock, M. Holden, D. Wilson, T. D. Read, J. van den Elsen, N. K. Priest, E. J. Feil, L. D. Hurst, E. Josefsson, and R. C. Massey. Genome Res. 2014, **24** (5), 839–849
6. S. Castillo-Ramírez, J. Corander, P. Marttinen, M. Aldeljawi, W. P. Hanage, H. Westh, K. Boye, Z. Gulay, S. D. Bentley, J. Parkhill, M. T Holden and E. J. Feil. Genome Biol. 2012, **13**, R126
7. K. Chua, T. Seemann, P. F. Harrison, J. K. Davies, S. J. Coutts, H. Chen, V. Haring, R. Moore, B. P. Howden, T. P. Stinear. J. Bacteriol., 2010, **192**, 5556–5557
8. M. Laabei, A-C. Uhlemann, F. D. Lowy, E. D. Austin, M. Yokoyama, K. Ouadi, E. Feil, H. A. Thorpe, B. Williams, M. Perkins, S. J. Peacock, S. R. Clarke, J. Dordel, M. Holden, A. A. Votintseva, R. Bowden, D. W. Crook, B. C. Young, D. J. Wilson, M. Recker, R. C. Massey. PLoS Biol., 13 (9), e1002229, DOI: 10.1371/journal.pbio.1002229
9. A-C. Uhlemann, J. Dordel, J. R. Knox, K. E. Raven, J. Parkhill, M. T. G. Holden, S. J. Peacock, and F. D. Lowy. Proc. Natl. Acad. Sci. U. S. A., 2014, **111** (18), 6738-6743
10. Y-Q. Zhang, S-Xi. Ren, H-L. Li, Y-X. Wang, G. Fu, J. Yang, Z-Q. Qin, Y-G. Miao, W-Y. Wang, R-S. Chen, Y. Shen, Z. Chen, Z-H. Yuan, G-P. Zhao, D. Qu, A. Danchin, Y-M. Wen. Mol. Microbiol., 2003, **49** (6), 1577–1593
11. Culture collections: Staphylococcus epidermidis. https://www.phe-culturecollections.org.uk/products/bacteria/detail.jsp?refId=NCTC%2013360&collection

=nctc. Date accessed: 02/10/2020.

12. Staphylococcus epidermidis (Winslow and Winslow) Evans (ATCC® 12228™). https://www.lgcstandards-atcc.org/products/all/12228.aspx?geo_country=gb. Date accessed: 02/10/2020.
13. Biomodification of Abiotic Surfaces for the Prevention of Hospital-Associated Infection. PhD Thesis. University of Bath. 2017.
14. S. R. Gill, D. E. Fouts, G. L. Archer, E. F. Mongodin, R. T. DeBoy, J. Ravel, I. T. Paulsen, J. F. Kolonay, L. Brinkac, M. Beanan, R. J. Dodson, S. C. Daugherty, R. Madupu, S. V. Angiuoli, A. S. Durkin, D. H. Haft, J. Vamathevan, H. Khouri, T. Utterback, C. Lee, G. Dimitrov, L. Jiang, H. Qin, J. Weidman, K. Tran, K. Kang, I. R. Hance, K. E. Nelson, C. M. Fraser. J. Bacteriology, 2005, **187**, 2426–2438
15. Staphylococcus epidermidis (Winslow and Winslow) Evans (ATCC® 35984™). https://www.lgcstandards-atcc.org/Products/All/35984.aspx?geo_country=gb. Date accessed: 02/10/2020.
16. J. Klockgether, A. Munder, J. Neugebauer, C. F. Davenport, F. Stanke, K. D. Larbig, S. Heeb, U. Schöck, T. M. Pohl, L. Wiehlmann and B. Tümmler. J. Bacteriology. 2010, **192**, 1113–1121
17. B. W. Holloway. J. Gen. Microbiol. 1955, **13**, 572-581
18. N. T. Thet, J. Mercer-Chalmers, R. J. Greenwood, A. E. R. Young, K. Coy, S. Booth, A. Sack, and A. T. A. Jenkins. ACS Sens. 2020, **5** (8), 2652–2657
19. Culture Collections: Escherichia coli. https://www.phe-culturecollections.org.uk/products/bacteria/detail.jsp?refId=NCTC%2010418&collection=nctc. Date accessed: 02/10/2020.
20. N. Savorya, J. Nzakizwanayo, K. Abe, W. Yoshida, S. Ferri, C. Dedi, B. V. Jones, K. Ikebukuroa. J. Microbiol. Methods. 2014, **104**, 94-100
21. S. G. N. Grant, J. Jesse, F. R. Bloom, and D. Hanahan. Proc. Natl. Acad. Sci. U.S.A., 1990,
87, 4645-4649
22. A. E. Jacob and S. J. Hobbs. *J. Bactriol.* 1974, **117**, 360-372
23. A. A. Miles, S. S. Misra, and J. O. Irwin, *J. Hyg*,. 1938, **38**, 732–749
24. Clinical and Laboratory Standards Institute (CLSI) (2019) Performance standards for antimicrobial susceptibility testing. 29th informational supplement, MS100-S29. CLSI, Pennsylvania, USA

25. N. T. Thet, D. R. Alves, J. E. Bean, S. Booth, J. Nzakizwanayo, A. E. R. Young, B. V. Jones and A. T. A. Jenkins. *ACS Appl. Mater. Interfaces*. 2016, 8, 24, 14909–14919
26. M. Roth-Konforti, O. Green, M. Hupfeld, L. Fieseler, N. Heinrich, J. Ihssen, R. Vorberg, L. Wick, U. Spitz, and D. Shabat. *Angew. Chem. Intl. Ed.*, 2019. **131**(30), 10469-10475.
27. S. Das, J. Ihssen, L. Wick, U. Spitz, and D. Shabat, *Chem. Eur. J.*. 2020. **26**, 3647 –3652
28. S. Maity, X. Wang, S. Das, M. He, L.W. Riley, and N. Murthy, *Chem. Commun.*, 2020, **56** (24), 3516-3519.
29. S. Jahanshahi-Anbuhi, B. Kannan, K. Pennings, M. M. Ali, V. Leung, K. Giang, J. Wang, D. White, Y. Li, R.H. Pelton, and J. D. Brennan, *Lab on a Chip*, 2017, **17**(5), 943-950.
30. N.S.K. Gunda, R. Chavali, and S. K. Mitra, *Analyst*, 2016. **141**(10), 2920-2929.
31. D. Wildeboer, L. Amirat, R.G. Price, and R. A. Abuknesha, *Water research*, 2010, **44** (8), 2621-2628.
32. S. Langlet, F. Beaupère, G. Contant, and J. M. Scheftel, *FEMS microbiology letters*, 1999. **170** (1), pp.229-235.
33. H. J. Kim, C. Kwon, B. S. Lee, and H. Noh, *Analyst*, 2019. **144** (7), 2248-2255.
